# First Class of Phosphorus Dendritic Compounds Containing β-Cyclodextrin Units in the Periphery Prepared by CuAAC

**DOI:** 10.3390/molecules25184034

**Published:** 2020-09-04

**Authors:** Kendra Sorroza-Martínez, Israel González-Méndez, Mireille Vonlanthen, Kathleen I. Moineau-Chane Ching, Anne-Marie Caminade, Javier Illescas, Ernesto Rivera

**Affiliations:** 1 Instituto de Investigaciones en Materiales, Universidad Nacional Autónoma de México, Circuito Exterior, Ciudad Universitaria, Mexico City CP 04510, Mexico; kendraivn17@gmail.com (K.S.-M.); mireille.vonlanthen@gmail.com (M.V.); 2 CNRS, LCC (Laboratoire de Chimie de Coordination), 205 route de Narbonne, BP 44099, F-31077 Toulouse, CEDEX 4, France; kathleen.chane@lcc-toulouse.fr (K.I.M.-C.C.); anne-marie.caminade@lcc-toulouse.fr (A.-M.C.); 3 LCC-CNRS, Université de Toulouse, CNRS, 31077 Toulouse, France; 4 Tecnológico Nacional de México/Instituto Tecnológico de Toluca, Avenida Tecnológico S/N Col. Agrícola Bellavista, Metepec CP 52140, Mexico; fillescasm@toluca.tecnm.mx

**Keywords:** phosphorus dendritic compounds, β-cyclodextrin, CuAAC reaction

## Abstract

A new class of phosphorus dendritic compounds (PDCs) having a cyclotriphosphazene (P_3_N_3_) core and decorated with six β-cyclodextrin (βCD) units, named P_3_N_3_-[O-C_6_H_4_-O-(CH_2_)_n_-βCD]_6_, where *n* = 3 or 4 was designed, and the synthesis was performed using copper (I) catalyzed alkyne-azide cycloaddition (CuAAC). To obtain the complete substitution of the P_3_N_3_, two linkers consisting of an aromatic ring and an aliphatic chain of two different lengths were assessed. We found that, with both linkers, the total modification of the periphery was achieved. The two new obtained dendritic compounds presented a considerably high water solubility (>1 g/mL). The compounds comprised in this new class of PDCs are potential drug carrier candidates, since the conjugation of the βCD units to the P_3_N_3_ core through the primary face will not only serve as surface cover but, also, provide them the faculty to encapsulate various drugs inside the βCDs cavities.

## 1. Introduction

The use of nanomedicine confers a substantial potentiality for drug delivery and targeted release, as it increases the safety by reducing toxic effects in nontargeted organs and tissues [1,2,3]. In recent years, many researchers have focused on the development of dendrimers, since they have distinctive properties, such as monodispersity, a large number of easily available functional groups on the surface and an extraordinary capability to encapsulate host molecules within their hydrophobic environment, making them ideal nanocarriers for the targeted delivery (with or without ligand) of therapeutic and diagnostic agents [4,5,6].

Dendrimers are well-defined hyper-branched three-dimensional macromolecules. Structurally, dendrimers have a core from which branches (or arms) are derived and end with multiple peripheral groups that determine their macroscopic properties [7]. Dendrimers can be constructed starting from the core towards the periphery (divergent synthesis) or through a top-down approach, from the appropriate outer residues (convergent synthesis) [8]. It is clear that, when designing a new dendrimer, the chemical composition of the core, branches and peripheral groups will have crucial implications for enhancing possible biomedical applications, either by themselves or as nanocarriers [9].

Among the diverse existing types of dendrimers, phosphorus containing dendrimers, (i.e., dendrimers having phosphorus derivatives in their structure) play a special role in applications such as drug delivery, gene therapy and biomaterials, among others [10,11].

In 1994, Caminade et al. described the first method for the synthesis of high-generation phosphorus dendrimers (PDs), which is still the most widely used, since it allows numerous changes at the level of the core, branches or terminal groups, conferring biocompatible functions for the use in biological applications [12]. In this way, PD synthesis can be started from different cores; however, hexachlorocyclotryphosphazene (P_3_N_3_Cl_6_) is the most used core, since it provides twice the number of final groups, compared with thiophosphoryl chloride (P(S)Cl_3_) [10]. Using the P_3_N_3_Cl_6_ core, modifications to the terminal groups of the PDs can be achieved with different molecules in order to improve the biocompatible properties. One of the strategies used is the conjugation with different types of carbohydrates to improve the aqueous solubility and correlate the structures with the impacts on biological behaviors [13,14]. For instance, the construction of PDs with mannose residues in its periphery was employed as a strategy for the treatment of lung inflammation [15]. Furthermore, PDs conjugated with mannopyranoside have been used as multivalent carbohydrate protein-binding recognition probes [16]. Finally, PDs were used as noncovalent carriers of aminolactitol carbohydrate for treatment against the HIV virus [17].

With the focus of developing carbohydrate-conjugated dendrimers, diverse modifications of poly(amidoamines) (PAMAM) dendrimers with cyclodextrin molecules (CDs) have been reported for multiple applications [18,19,20,21]; however, this strategy has not yet been reported for PDs. CDs are cyclic oligosaccharides, composed of six or more glucopyranose units linked by α (1-4)-glucosidic bonds. Typical native CDs contain six, seven or eight glucose units and are named α-, β- and γ-, respectively. Due to the lack of free rotations between glucose units, CDs present a truncated cone shape and a hydrophobic internal cavity. β-cyclodextrin (βCD) is the most studied and frequently used CD, due to its low cost, availability and ability to encapsulate a wide range of molecules in its internal cavity. The variable reactivity of the hydroxyl groups and the remarkable encapsulation properties that can modify and/or improve the physical, chemical and biological characteristics of host molecules make it the perfect candidate to be used as building blocks in the construction of drug delivery systems [22,23,24].

Synthesis mediated by copper-catalyzed azide-alkyne cycloaddition (CuAAC) to produce 1,4-disubstituted 1,2,3-triazoles has been demonstrated to be effective in the design of new dendrimers. CuAAC coupling confers high specificity, mild reaction conditions and quantitative synthetic yields [25]. The physicochemical properties of the triazole ring are particularly favorable when added to structures with biological applications, since triazole units act as rigid bonds. Unlike amides, the triazole ring cannot be hydrolytically cleaved, oxidized or reduced [26].

For all these reasons, in this work, we propose the design, synthesis and characterization of a first class of phosphorus dendritic compounds (PDCs) decorated with βCD units, using aromatic rings and aliphatic chains as spacers P_3_N_3_-[O-C_6_H_4_-O-(CH_2_)_n_-βCD]_6_ (*n* = 3 or 4). We selected P_3_N_3_ as the core, and in the first step, alkyne groups were added to the periphery. Finally, the grafting of βCD units was carried out by the CuAAC reaction. The grafting of βCD units on its primary side will allow the secondary face to be available for the formation of inclusion complexes, so that these new PDCs are potential candidates as drug nanocarriers and may have applications in nanomedicine.

## 2. Results and Discussion

### 2.1. Synthesis

**Scheme 1:**
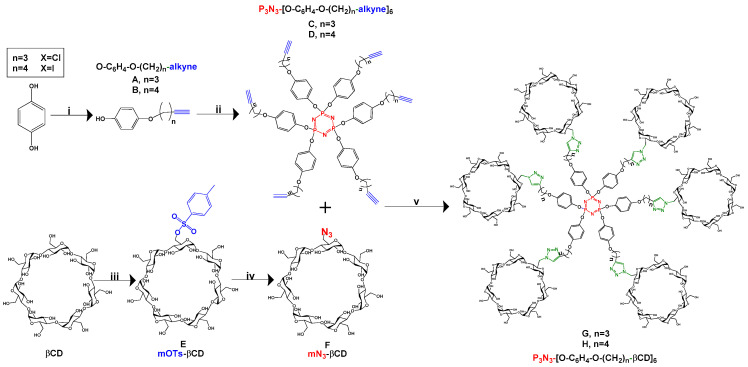
Synthesis of P_3_N_3_-[O-C_6_H_4_-O-(CH_2_)_n_-βCD]_6_ (*n* = 3 or 4) phosphorus dendritic compounds (PDCs). Conditions: (i) K_2_CO_3_, X-alkyne, *N,N*-dimethylformamide (DMF) anhydrous, 72 °C, 36 h. (ii) (**A**,**B**), Cs_2_CO_3_, P_3_N_3_Cl_6_, tetrahydrofuran anhydrous (THF) anhydrous, 7 days, room temperature (RT). (iii) Ts_2_O/NaOH, H_2_O, 2 h, RT. (iv) NaN_3_, DMF anhydrous, 80 °C, 48 h. (v) mN_3_-βCD, CuSO_4_•5H_2_O, H_2_Asc, dimethylsulfoxide (DMSO):H_2_O (7:1), 80 °C, 7 days.

The synthetic route of intermediates HO-C_6_H_4_-O-(CH_2_)_n_-alkyne (where *n* = 3 or 4) is shown in Scheme 1. The intermediates **A** and **B** were prepared via a Williamson etherification reaction between the hydroquinone and the respective X-alkynes (X = Cl for *n* = 3 and I for *n* = 4), using K_2_CO_3_ as a base and anhydrous *N,N*-dimethylformamide (DMF) as the solvent at 72 °C. Mono and difunctionalized products were obtained, and they could be separated by column chromatography. Subsequently, the fraction corresponding to the monofunctionalized product was recrystallized in a cold/hot hexane to give the compounds **A** and **B** [27].

The synthetic route of intermediates P_3_N_3_-[O-C_6_H_4_-O-(CH_2_)_n_-alkyne]_6_ (*n* = 3 or 4) **C** and **D** is shown in Scheme 1. The preparation of the intermediates was achieved, as previously reported in the literature but using phenols with a different substitution pattern [28]. The reaction proceeded through a substitution reaction of the Cl atoms attached to the P_3_N_3_ core; this substitution was carried out in basic and anhydrous medium using intermediates **A** and **B** to obtain compounds **C** and **D**, respectively.

The next step in the synthetic route was the formation of mOTs-βCD (**E**), followed by mN_3_-βCD (**F**), which is shown in Scheme 1. For the synthesis of mOTs-βCD, the most efficient technique reported in the literature was employed to modify a single position of the native βCD [29], with minimal differences in the purification of the final product. It is worth noticing that this synthetic step was crucial in the design of our PDCs. In particular, the βCD tosylation method provided high yields compared to other previously reported methods [30,31]. Then, the tosyl group of compound **E** was replaced by an azide group through a nucleophilic substitution reaction to give compound **F** [32].

Once the necessary intermediates, alkynes (P_3_N_3_-[O-C_6_H_4_-O-[CH_2_]_n_-alkyne]_6_) and azide (mN_3_-βCD) were obtained, the CuAAC reaction was carried out, as shown in Scheme 1. According to the design of these novel PDCs, we used two linkers with different lengths of the aliphatic chain to assess whether this factor had an influence on the complete functionalization of all positions of the P_3_N_3_ core. To carry out the CuAAC reaction, we used a Cu(I) catalyst synthesized in situ, with CuSO_4_ as the copper source and H_2_Asc as a reducing agent, using dimethylsulfoxide (DMSO) as the ideal solvent, since it solubilizes the azide and the respective alkynes. To ensure the complete functionalization of the six core groups of P_3_N_3_, an excess of mN_3_-βCD was added. The final PDCs **G** and **H** were purified by size exclusion chromatography, using water as the eluent, and were obtained in high purity and with yields of >50%. We found that the length of the linker did not affect the complete functionalization of P_3_N_3_ with the βCD units, since all six positions of the P_3_N_3_ core were functionalized with both proposed linkers.

### 2.2. Characterization

The full characterization of all the synthetic intermediates and final compounds was carried out. Regarding intermediates **A** and **B**, in the ^1^H-NMR spectra (Figures S1 and S4 are available in Supplementary Materials (SM)) in DMSO-*d*_6_, it is possible to observe the signal corresponding to the proton of the alkyne group at 2.80 and 2.78 ppm, respectively. Moreover, the signal of the phenol proton appeared between 8.90 and 8.88 ppm. The structure of intermediates **A** and **B** was also confirmed by the ^13^C-NMR spectra (Figures S2 and S5 are available in SM), in which the signals corresponding to the carbons of the alkyne group appeared at 84.56 and 72.34 ppm and at 83.42 and 72.19 ppm, respectively. The structure of these intermediates was also confirmed by mass spectrometry using the Direct Analysis in Real Time (DART) technique (Figures S3 and S6 are available in SM). The molecular ions appeared at 177 *m*/*z* (**A**) and 191 *m*/*z* (**B**), which correspond to the molecular weights of the proposed compounds.

The presence of phosphorus in the core allowed simple monitoring by ^31^P-NMR; furthermore, this allowed to verify the completion of the reactions in each synthesis step, as well as the integrity of the entire structure [33]. For the characterization of the intermediates **C** and **D**, a single signal corresponding to the complete functionalization was observed in the ^31^P-NMR spectrum (Figures S9 and S13 are available in SM). Furthermore, the complete functionalization of the phosphorous core was confirmed with the rest of the characterization. In the ^1^H-NMR spectrum (Figures S7 and S11 are available in SM), the disappearance of the signal corresponding to the phenol proton present in intermediates **A** and **B**, as mentioned above, is clearly observed due to its grafting on P_3_N_3_. The structure of **C** and **D** was also confirmed by ^13^C-NMR (Figures S8 and S12 are available in SM) and Matrix-Assisted Laser Desorption/Ionization Time-Of-Flight (MALDI-TOF) mass spectrometry (Figures S10 and S14 ESI), where the molecular ions appeared at 1187.623 *m*/*z* and 1271.794 *m*/*z*, which correspond to the molecular weights of compounds **C** and **D**, respectively. The structures of the compounds P_3_N_3_-[*O*-C_6_H_4_-*O*-(CH_2_)_n_-alkyne]_6_ (where *n* = 3 or 4) were confirmed by all the characterization data.

**Figure 1:**
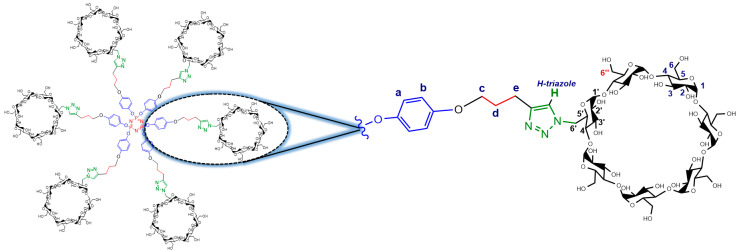
Assignation of the protons in the NMR of P_3_N_3_-[*O*-C_6_H_4_-*O*-(CH_2_)_3_-βCD]_6_ dendritic compounds (PDCs).

**Figure 2:**
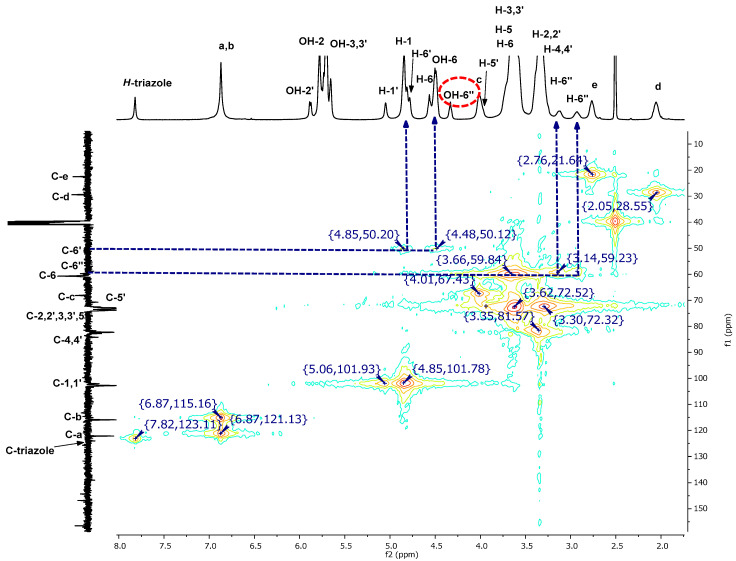
2D NMR Heteronuclear Multiple-Quantum Correlation (HMQC) spectra of P_3_N_3_-[*O*-C_6_H_4_-*O*-(CH_2_)_3_-βCD]_6_ PDCs in DMSO-*d*_6_.

**Figure 3:**
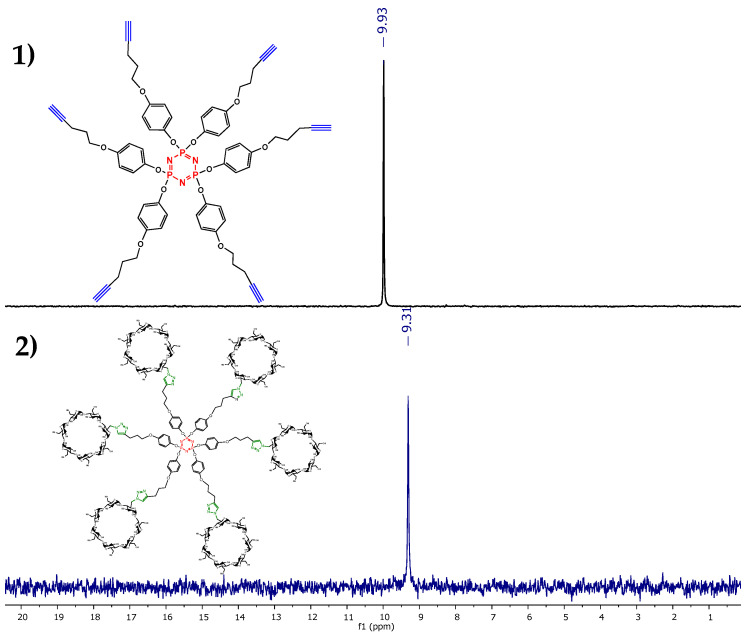
Split of ^31^P-NMR in DMSO-*d*_6_ of (**1**) P_3_N_3_-[*O*-C_6_H_4_-*O*-(CH_2_)_3_-alkyne]_6_ (compound **C**) and (**2**) P_3_N_3_-[*O*-C_6_H_4_-*O*-(CH_2_)_3_-βCD]_6_ (PDC **G**).

The full characterization of two new P_3_N_3_-[*O*-C_6_H_4_-*O*-(CH_2_)_n_-βCD]_6_ (**G**) and (**H**) PDCs using NMR techniques (^1^H-,^13^C-NMR and 2D NMR Heteronuclear Multiple-Quantum Correlation (HMQC) and Homonuclear Correlation Spectroscopy (COSY)) (Figures S15–S17 and S19–S22 are available in SM) was carried out in DMSO-*d*_6_. The classical signals of the linkers and native βCD units are in agreement with those reported in previous works [17,26]. Moreover, it was possible to observe the differentiation for some protons of the modified glucopyranose unit of the βCD (see Figure 1). The signals of protons H-1’, H-5’ and, particularly, H-6’ appeared at a lower field than those of H-1, H-5 and H-6 of the nonfunctionalized subunits, due to the change in their chemical environment after the CuAAC reaction. In the same way, it was possible to identify the H-6’’ diastereotopic protons, corresponding to one -CH_2_-OH fragment contiguous to the substituted one at 3.12 and 2.94 ppm (see HMQC in Figure 2). Therefore, H-6’’ and the adjacent OH-6” on the primary face (around 4.33 ppm) appeared significantly upfield-shifted in comparison to their respective analogs H-6 and OH-6 because of the change in chemical environment due to the neighboring substitution. In this step, ^31^P-NMR was used to assess the completion of the reactions and to assure the complete functionalization of the six positions of P_3_N_3_. This was confirmed by the appearance of a single signal (at 9.31 ppm) in the ^31^P-NMR spectrum of P_3_N_3_-[*O*-C_6_H_4_-*O*-(CH_2_)_n_-βCD]_6_ (where *n* = 3, compound **G**). This signal exhibited an upfield shift compared to the signal corresponding to P_3_N_3_-[O-C_6_H4-O-(CH_2_)_n_-alkyne]_6_ (where *n* = 3, compound **C**) (at 9.93 ppm), because P_3_N_3_ is protected by βCD molecules (Figure 3). The same behavior was observed in the ^31^P-NMR spectrum (Figure S24 is available in SM) of P_3_N_3_-[*O*-C_6_H_4_-*O*-(CH_2_)_n_-βCD]_6_ (where *n* = 4, compound **H**). Finally, the structures of these new dendrimers were corroborated by MALDI-TOF mass spectrometry (Figures S18 and S23 are available in SM). The molecular ions appeared mainly at 8169.59 *m*/*z* and at 8254.30 *m*/*z*, corresponding to the molecular weights of the PDCs **G** and **H**, respectively. Furthermore, the signals at 7034.596 *m*/*z* and 7120.029 *m*/*z* for compounds **G** and **H**, respectively, correspond to partial ionization, since complete ionization of the molecule is complex when using this technique. This difficulty has been previously reported for other phosphorus dendrimers [34].

### 2.3. Determination of Water Solubility for P_3_N_3_-(O-C_6_H_4_-O-(CH_2_)_n_-βCD)_6_ PDCs

Among all the special properties of dendrimers, their high solubility makes an important difference compared to linear polymers. Differences in solubility between hyper-branched and linear polymers can be of several orders of magnitude, up to 10^6^ times, in some cases [35]. Among all the solvents used to dissolve dendrimers, water is especially important when considering biological applications. In recent years, the importance of water-soluble phosphorous-containing dendrimers has increased. In most cases, the water solubility of these compounds was dependent mainly on the reactivity of terminal groups in the periphery [36]. Therefore, the determination of the water solubility of our new PDCs was carried out, according to a method previously reported in the literature [37]. It was found that, for both PDCs, the solubility in water was >1g/mL. These results represent a considerably higher solubility compared to the solubility of native βCD (18.5 mg/mL) and of other commercial βCD derivatives, such as sulfobutylether-βCD (>500 mg/mL), *O*-methyl-βCD (>500 mg/mL) and 2-hydroxypropyl-βCD (>600 mg/mL) [38]. Water-soluble phosphorous-containing dendrimers with cationic or anionic end groups have been reported; nevertheless, their water solubility was dependent on the pH [17,35]. On the contrary, the advantage of our new P_3_N_3_-[*O*-C_6_H_4_-*O*-(CH_2_)_n_-βCD]_6_ PDCs (*n* = 3 and 4) is that their solubility in water is conferred by the βCD units in standard conditions. 

## 3. Materials and Methods

### 3.1. General Notes

All starting materials were commercially available reagent grade and were used without any further purification. Hexachlorocyclotriphosphazene (P_3_N_3_Cl_6_), 5-chloro-1-pentyne, 6-iodo-1-hexyne, hydroquinone, β-cyclodextrin (βCD), *p*-toluenesulfonyl chloride (Cl-Ts), *p*-toluenesulfonic acid (OH-Ts), *N,N*-dimethylformamide anhydrous (DMF), potassium carbonate (K_2_CO_3_), cesium carbonate (Cs_2_CO_3_), dimethylsulfoxide (DMSO), ascorbic acid (H_2_Asc), Bio-Gel P-10 medium from Bio-Rad (Hercules, CA, USA), potassium iodide (KI), sodium azide (NaN_3_), sodium hydroxide (NaOH), copper sulfate pentahydrate (CuSO_4_•5H_2_O), diisopropyl ether, dichloromethane (CH_2_Cl_2_), ethyl acetate (EtOAc), tetrahydrofuran anhydrous (THF), hexane (HEX), methanol (MeOH), ethanol (EtOH) and acetone (CH_3_COCH_3_) were purchased from Sigma-Aldrich (St. Louis, MO, USA).

### 3.2. Synthetic Procedures

#### 3.2.1. Synthesis of HO-C_6_H_4_-*O*-(CH_2_)_n_-alkyne (*n* = 3 and 4) **A** and **B**

The synthesis of the intermediates **A** and **B** was carried out according to a previously reported procedure, with some modifications [27,28]. A dry hydroquinone solution (24.03 mmol) in DMF (250 mL) was refluxed for 30 min at 72 °C, K_2_CO_3_ (30.04 mmol) was added and the mixture was refluxed for 1 h. To this mixture, alkyne (12.02 mmol) was added dropwise over 2 h. The resulting mixture was refluxed for 36 h, then cooled to 25 °C and filtered. The filtrate was evaporated under reduced pressure. The resulting brown oil was dissolved in CH_2_Cl_2_ (150 mL), and the solution was extracted with water (3 × 50 mL), the organic phase was dried with anhydrous Na_2_SO_4_ and the solvent was evaporated. The crude product was composed of a mixture of unreacted hydroquinone, the monofunctionalized and the difunctionalized products, which were separated by column chromatography on silica gel using hexanes:EtOAc (8:2). Once the fraction corresponding to the monofunctionalized product was obtained, it was recrystallized from hot/cold hexane, and the final product was recovered by filtration; the solid was left to dry overnight under vacuum. HO-C_6_H_4_-*O*-(CH_2_)_3_-alkyne was obtained as a beige solid (7.17 mmol, 29%). HO-C_6_H_4_-*O*-(CH_2_)_4_-alkyne was obtained as a yellow solid (7.88 mmol, 66%).

*HO-C_6_H_4_-O-(CH_2_)_3_-alkyne.*^1^H-NMR (400 MHz, DMSO-*d*_6_, δ ppm): 8.90 (s, 1H, PhOH), 6.76 (d, *J* = 9 Hz, 2H, H*a*), 6.68 (d, *J* = 9 Hz, 2H, H*b*), 3.93 (t, *J* = 2.4 Hz, 2H, H*c*), 2.80 (t, *J* = 2.4 Hz, 1H, C≡C-H), 2.31 (t, *J* = 2.4 Hz, 2H, H*e*), 1.86 (t, *J* = 2.4 Hz, 2H, H*d*); ^13^C-DEPTQ NMR (101 MHz, DMSO-*d*_6_, δ ppm): 152.01, 116.50, 116.20, 84.56, 72.34, 67.15, 28.67, 15.29. DART-MS: 177 *m*/*z* (M+H)^+^; 178 *m*/*z* (M + 2H)^+^.

*HO-C_6_H_4_-O-(CH_2_)_4_-alkyne.*^1^H-NMR (400 MHz, DMSO-*d*_6_, δ ppm): 8.88 (s, 1H, PhOH), 6.75 (d, *J* = 9 Hz, 2H, H*a*), 6.67 (d, *J* = 9 Hz, 2H, H*b*), 3.88 (t, *J* = 2.4 Hz, 2H, H*c*), 2.78 (t, *J* = 2.4 Hz, 1H, C≡C-H), 2.22 (t, *J* = 2.4 Hz, 2H, H*f*), 1.75 (t, *J* = 2.4 Hz, 2H, H*d*), 1.58 (t, *J* = 2.4 Hz, 2H, H*e*); ^13^C-DEPTQ NMR (101 MHz, DMSO-*d*_6_, δ ppm): 152.19, 151.88, 116.24, 116.14, 83.42, 72.19, 68.10, 28.75, 25.49, 18.26. DART-MS: 191 *m*/*z* (M)^+^; 192 *m*/*z* (M + H)^+^.

#### 3.2.2. Synthesis of P_3_N_3_-[O-C_6_H_4_-O-(CH_2_)_n_-alkyne]_6_ (*n* = 3 and 4) **C** and **D**

The synthesis of intermediates **C** and **D** was carried out according to a previously reported procedure, with some modifications [39]. A solution of intermediate **A** or **B** (2.84 mmol) in dry THF (25 mL) was stirred for 20 min. Cs_2_CO_3_ (5.68 mmol) was added to this solution and stirred for 1 h. Afterwards, P_3_N_3_Cl_6_ (0.32 mmol) was added to the reaction mixture, and it was left stirring at room temperature for 7 days, until the reaction was complete, monitored by ^31^P-NMR. The reaction mixture was then centrifuged at 10,000 rpm for 20 min to remove inorganic salts, the supernatant was recovered and the solvent was evaporated under reduced pressure. The crude product contained fully functionalized P_3_N_3_-[*O*-C_6_H_4_-*O*-(CH_2_)_n_-alkyne]**_6_** and the unreacted monoalkynes, which were separated by column chromatography on silica gel using CH_2_Cl_2_ as the eluent. Once the fraction corresponding to the functionalized P_3_N_3_ was obtained, it was recrystallized from cold isopropyl ether. P_3_N_3_-[*O*-C_6_H_4_-*O*-(CH_2_)_n_-alkyne]_6_ (*n* = 3 or 4) were obtained as white solids (0.19 mmol, 59% and 0.12 mmol, 52% for **C** and **D**, respectively).

*P_3_N_3_-[O-C_6_H_4_-O-(CH_2_)_3_-alkyne]_6_.*^1^H-NMR (400 MHz, DMSO-*d*_6_, δ ppm): 6.80 (d, *J* = 9.2 Hz, 12H, H***a***), 6.76 (d, *J* = 9.2 Hz, 12H, H***b***), 4.00 (t, *J* = 2.5 Hz, 12H, H***c***), 2.81 (t, *J* = 2.41 Hz, 6H, C≡C-**H**), 2.34 (t, *J* = 2.41 Hz, 12H, H***e***), 1.90 (t, *J* = 2.41 Hz, 12H, H***d***); ^13^C-DEPTQ NMR (101 MHz, DMSO-*d*_6_, δ ppm): 156.34, 144.23, 122.21, 115.83, 84.36, 72.40, 67.11, 28.56, 15.31; ^31^P-NMR (162 MHz, DMSO-*d*_6_, *δ* ppm): s, 9.93. MALDI-TOF-MS: 1187.62 *m*/*z* (M)^+^.

*P_3_N_3_-[O-C_6_H_4_-O-(CH_2_)_4_-alkyne]_6_.*^1^H-NMR (400 MHz, DMSO-*d*_6_, δ ppm): 6.78 (d, *J* = 9.2 Hz, 12H, H*a*), 6.72 (d, *J* = 9.2 Hz, 12H, H*b*), 3.94 (t, *J* = 2.5 Hz, 12H, H***c***), 2.78 (t, *J* = 2.41 Hz, 6H, C≡C-H), 2.24 (t, *J* = 2.41 Hz, 12H, H***f***), 1.80 (m, 12H, H***d***); 1.61 (m, 12H, H***e***); ^13^C-DEPTQ NMR (101 MHz, DMSO-*d*_6_, δ ppm): 156.46, 144.19, 122.20, 115.77, 85.01, 72.17, 68.08, 28.64, 25.48,18.28; ^31^P-NMR (162 MHz, DMSO-*d*_6_, *δ* ppm): s, 10.01. MALDI-TOF-MS: 1271.79 *m*/*z* (M + H)^+^.

#### 3.2.3. Synthesis of 6-*O*-monotosyl-β-cyclodextrin (mOTs-βCD) **E**

Following the procedure previously reported [29], in a round-bottom flask, *p*-toluenesulfonic acid (0.0101 mol) and *p*-toluenesulfonyl chloride (0.039 mol) were dissolved in 50 mL of CH_2_Cl_2_ and stirred at room temperature for 12 h. Then, the reaction mixture was filtered, and the filtrate was concentrated under reduced pressure. The residue was recrystallized (3×) with cold hexane, and the product (Ts_2_O) was allowed to dry under vacuum. Afterwards, in a round-bottom flask, βCD (0.0051 mol) and Ts_2_O (0.0076 mol) were dissolved in 150 mL of H_2_O. The reaction mixture was stirred for 2 h at room temperature; after this time, a 2.5-M aqueous NaOH solution was added. The reaction mixture was stirred for 10 min. Subsequently, the mixture was filtered, and the pH of the filtrate was adjusted to 8 with a saturated solution of ammonium chloride to give a precipitate. The mixture was filtered, and the precipitate was recrystallized (3×) in acetone. The product **E** was obtained as a white solid (0.0021 mol, 42%).

^1^H-NMR (400 MHz, DMSO-*d*_6_, δ ppm): 7.75 (b, 2H) a, 7.44 (b, 2H) b, 5.83 (d, *J* = 6.4 Hz, 1H) OH2′, 5.78 (b, 6H) OH2, 5.71 (b, 7H) OH3, 4.84 (d, *J* = 3.9 Hz, 6H) H1, 4.76 (d, *J* = 3.9 Hz, 1H) H1′, 4.50 (m, 6H) OH6, 4.35 (m, 2H) H6′ab, 4.19 (m, 1H) H5′, 3.65 (m, 12 H) H6ab, 3.60 (b, 7H) H3, 3.51 (m, 7H) H5, 3.30 (m, 7H) H2, 3.22 (m, 7H) H_4_, 2.42 (d, 3H) c. ^13^C-NMR (100 MHz, DMSO-*d*_6_, δ ppm): 145.25, 133.09, 130.29, 128.03, 102.39, 101.74, 81.95, 81.21, 73.43, 73.16, 72.84, 72.48, 70.17, 69.36, 60.28, 21.62. MALDI-TOF-MS (*m*/*z*): 1311.591 (M + Na)^+^.

#### 3.2.4. Synthesis of 6-*O*-monoazido-β-cyclodextrin (mN_3_-βCD) **F**

Following a previously reported procedure [32], in a round-bottom flask, mOTs-βCD (0.0016 mol), NaN_3_ (0.005 mol) and KI (0.0008 mol) were dissolved in 8 mL of anhydrous DMF. The reaction mixture was stirred at 80 °C for 48 h. After this time, DMF was evaporated under reduced pressure, and the residue was recrystallized in a mixture, H_2_O:Acetone (1:1), and allowed to dry under vacuum. The product **F** was obtained as a white solid (0.0014 mol, 88%).

^1^H-NMR (400 MHz, DMSO-*d*_6_, δ ppm): 5.74 (m, 7H) OH2, 5.67 (m, 6H) OH3, 5.62 (d, *J* = 2.4 Hz, 1H) OH3′, 4.88 (d, *J* = 3.5 Hz, 1H) H1′, 4.83 (m, 6H) H1, 4.48 (m, 6H) OH6, 3.77 (m, 2H) H6′, 3.68 (m, 12H) H6, 3.60 (m, 7H) H3, 3.55 (m, 7H) H5, 3.39 (m, 7H) H4, 3.29 (m, 7H) H2. ^13^C-NMR (100 MHz, DMSO-*d*_6_, δ ppm): 102.38, 102.04, 83.41, 81.99, 73.50, 73.30, 72.85, 72.67, 72.46, 70.63, 60.30, 51.53. MALDI-TOF-MS (*m*/*z*): 1182.764 (M + Na)^+^.

#### 3.2.5. Synthesis of P_3_N_3_-[*O*-C_6_H_4_-*O*-(CH_2_)_n_-βCD]_6_ PDCs (*n* = 3 or 4) **G** and **H**

P_3_N_3_-(*O*-C_6_H_4_-*O*-(CH_2_)_n_-alkyne)_6_ (*n* = 3 or 4) (0.055 mmol) and mN_3_-βCD (0.496 mmol) were dissolved in DMSO (5 mL); this mixture was degassed by bubbling argon for 10 min. A solution of CuSO_4_•5H_2_O (0.055 mmol) in a DMSO:H_2_O mixture (0.5:0.5 mL), followed by a solution of H_2_Asc (0.165 mmol) in DMSO:H_2_O mixture (0.5:0.5 mL), were added dropwise over 5 min. The reaction mixture was heated to 80 °C with vigorous stirring and under the argon atmosphere for 7 days, until the reaction was complete, monitored by ^31^P NMR. At the end of this time, the reaction mixture was cooled and precipitated dropwise into cold acetone (200 mL). The precipitate was filtered under vacuum. The obtained solid was purified by size exclusion chromatography using Bio-Gel^®^ P-10 medium and water as the eluent. Once the corresponding fractions were obtained, they were lyophilized to evaporate the water. PDCs **G** and **H** were obtained as white solids (0.041 mmol, 61% and 0.048 mmol, 88% for **G** and **H**, respectively).

*P_3_N_3_-[O-C_6_H_4_-O-(CH_2_)_3_-βCD]_6_.*^1^H-NMR (400 MHz, DMSO-*d*_6_, *δ* ppm): 7.82 (s, 6H, *H*-triazole), 6.87 (s, 24H, H-a, b), 5.87 (m, 7H, OH-2’), 5.78 (s, 36H, OH-2), 5.70–5.66 (m, 42H, OH-3,3’), 5.05 (s, 7H, H-1’), 4.84–4.78 (m, 42H, H-1; H-6’), 4.56–4.50 (m, 36H, H-6’; OH-6), 4.33 (s, 6H, OH-6’’), 4.02 (m, 18H, H-c; H-5’), 3.65–3.59 (m, 138H, H-6, H-3,3’, H-5), 3.41–3.28 (m, 84 H, H4,4’, H-2,2’ overlapped with H_2_O), 3.12 (m, 7H, H-6’’), 2.94 (m, 7H, H-6’’), 2.77 (m, 12H, H-e), 2.05 (m, 12H, H-d); ^13^C-DEPTQ NMR (101 MHz, DMSO-*d*_6_, *δ* ppm): 156.55, 144.37, 123.37, 122.15, 115.93, 102.98, 102.02, 84.28, 82.36, 73.05, 72.88, 70.72, 68.13, 60.90, 59.80, 50.37, 29.42, 22.51. ^31^P-NMR (162 MHz, DMSO-*d*_6_, *δ* ppm): s, 9.31. MALDI-TOF-MS: 8169.59 *m*/*z* (M + K)^+^.

*P_3_N_3_-[O-C_6_H_4_-O-(CH_2_)_4_-βCD]_6_.*^1^H-NMR (400 MHz, DMSO-*d*_6_, *δ* ppm): 7.78 (s, 6H, *H*-triazole), 6.83 (s, 24H, H-a, b), 5.88 (m, 7H, OH-2’), 5.78-5.72 (s, 36H, OH-2), 5.70-5.63 (m, 42H, OH-3,3’), 5.05 (s, 7H, H-1’), 4.84-4.78 (m, 42H, H-1; H-6’), 4.58-4.49 (m, 36H, H-6’; OH-6), 4.34 (s, 6H, OH-6’’), 3.96 (m, 18H, H-c; H-5’), 3.65-3.59 (m, 138H, H-6, H-3,3’, H-5), 3.41-3.28 (m, 84 H, H4,4’, H-2,2’ overlapped with H_2_O), 3.12 (m, 7H, H-6’’), 2.94 (m, 7H, H-6’’), 2.66 (m, 12H, H-f), 1.76 (m, 24H, H-d, e); ^13^C-DEPTQ NMR (101 MHz, DMSO-*d*_6_, *δ* ppm): 156.56, 147.25, 123.86, 122.11, 115.96, 102.98, 101.98, 84.18, 82.80, 82.33, 81.62, 73.94, 72.91, 68.38, 60.73, 59.77, 50.79, 29.27, 26.29, 22.50. ^31^P-NMR (162 MHz, DMSO-*d*_6_, *δ* ppm): s, 9.42. MALDI-TOF-MS: 8254.30 *m*/*z* (M + K)^+^.

### 3.3. Characterization

^1^H-, ^13^C- and ^31^P-NMR measurements were carried out on a Bruker spectrometer (Bruker, Beerlika, MA, USA) (400 MHz) using DMSO-*d*_6_ as solvent. Chemical shifts are reported in ppm (δ), and the signals were described as singlet (s), doublet (d), triplet (t) and multiplet (m); coupling constants (*J*) were reported in Hz. DART and MALDI-TOF mass measurements were performed on a JEOL JMS-AX505-HA instrument (Peabody, MA, USA) and on a Bruker Daltonics Flex Analysis instrument (Bruker, Beerlika, MA, USA), respectively. Matrices of 2,5-dihydroxybenzoic acid (DHB), dithranol (DIT) and 2-acetylphloroglucinol (THAP) were used for MALDI-TOF.

### 3.4. Determination of Water Solubility for P_3_N_3_-[O-C_6_H_4_-O-(CH_2_)n-βCD]_6_ PDCs

The determination of the solubility of the P_3_N_3_-[O-C_6_H_4_-*O*-(CH_2_)_n_-βCD]_6_ PDCs was carried out according to a method previously reported by Jozwiakowski and Connors [36], with modifications. One gram of the compound was placed in three amber vials of 5 mL with a screw cap, and 1 mL of water was added. The vials were sealed with parafilm to avoid water evaporation. They were stirred in an oil bath at a constant temperature of 25 ± 0.01 °C for 48 h. The supernatant was separated from the solid phase by filtration through a Milli-Q membrane (pore size of 0.45 µm) by injection of the mixture into disposable plastic syringes (Franklin Lakes, NJ, USA) of 3 mL at 25 °C. The supernatant of each sample was placed in three different vials. The samples were lyophilized for 48 h, and the obtained solids were weighed on a scale with an uncertainty of ±0.0001 g.

## 4. Conclusions

In this work, two novel phosphorus dendritic compounds (PDCs) containing a P_3_N_3_ as the core and βCD units as terminal groups were designed. The synthesis was carried out using the CuAAC reaction, giving high yields and products that were purified by a simple method. The complete functionalization of the P_3_N_3_ core of the new molecules was carried out using two aliphatic chains of different lengths as the linker. We found that there was no significant impact of the aliphatic chain length, since, with both linkers, the functionalization was complete. This is the first report for PDCs of the type P_3_N_3_-[O-C_6_H_4_-O-(CH_2_)_n_-βCD]_6_ arising from a novel design of water-soluble PDCs. The potential use of these new dendrimers as drug carriers for biomedical applications is currently under study.

## Supplementary Materials

The following are available online: NMR & MS spectrum of the compounds.
