# Supplementary material for: First Class of Phosphorus Dendritic Compounds Containing β-Cyclodextrin Units in the Periphery Prepared by CuAAC
Source: Molecules. 2020 Sep 4;25(18):4034. doi: 10.3390/molecules25184034 (PMC7570757; doi:10.3390/molecules25184034)
Supplement: Supplementary file 1 [file molecules-25-04034-s001.pdf]

# *Supplementary Information*

**Analysis data of**


**Figure S1.** $^{1}H$-NMR spectrum of HO-$C_6H_4$-O-$(CH_2)_3$-alkyne. p3

**Figure S2.** $^{13}C$-NMR spectrum of HO-$C_6H_4$-O-$(CH_2)_3$-alkyne. p3

**Figure S 3.** DART spectrum of HO-$C_6H_4$-O-$(CH_2)_3$-alkyne. p4

**Figure S4.** $^{1}H$-NMR spectrum of HO-$C_6H_4$-O-$(CH_2)_4$-alkyne. p4

**Figure S5.** $^{13}C$-NMR spectrum of HO-$C_6H_4$-O-$(CH_2)_4$-alkyne. p5

**Figure S6.** DART spectrum of HO-$C_6H_4$-O-$(CH_2)_4$-alkyne. p5

**Figure S7.** $^{1}H$-NMR spectrum of $P_3N_3$-[O-$C_6H_4$-O-$(CH_2)_3$-alkyne]$_6$. p6

**Figure S8.** $^{13}C$-NMR spectrum of $P_3N_3$-[O-$C_6H_4$-O-$(CH_2)_3$-alkyne]$_6$. p6

**Figure S9.** $^{31}P$-NMR spectrum of $P_3N_3$-[O-$C_6H_4$-O-$(CH_2)_3$-alkyne]$_6$. p7

**Figure S10.** MALDI spectrum of $P_3N_3$-[O-$C_6H_4$-O-$(CH_2)_3$-alkyne]$_6$. p7

**Figure S11.** $^{1}H$-NMR spectrum of $P_3N_3$-[O-$C_6H_4$-O-$(CH_2)_4$-alkyne]$_6$. p8

**Figure S12.** $^{13}C$-NMR spectrum of $P_3N_3$-[O-$C_6H_4$-O-$(CH_2)_4$-alkyne]$_6$. p8

**Figure S13.** $^{31}P$-NMR spectrum of $P_3N_3$-[O-$C_6H_4$-O-$(CH_2)_4$-alkyne]$_6$. p9

**Figure S14.** MALDI spectrum of $P_3N_3$-[O-$C_6H_4$-O-$(CH_2)_4$-alkyne]$_6$. p9

**Figure S15.** $^{1}H$-NMR spectrum of $P_3N_3$-[O-$C_6H_4$-O-$(CH_2)_3$-βCD]$_6$. p10

**Figure S16.** $^{13}C$-NMR spectrum of $P_3N_3$-[O-$C_6H_4$-O-$(CH_2)_3$-βCD]$_6$. p10

**Figure S17.** 2D NMR COSY spectrum of $P_3N_3$-[O-$C_6H_4$-O-$(CH_2)_3$-βCD]$_6$. p11

**Figure S18.** MALDI spectrum of $P_3N_3$-[O-$C_6H_4$-O-$(CH_2)_3$-βCD]$_6$. p12

**Figure S19.** $^{1}H$-NMR spectrum of $P_3N_3$-[O-$C_6H_4$-O-$(CH_2)_4$-βCD]$_6$. p12

**Figure S20.** $^{13}C$-NMR spectrum of $P_3N_3$-[O-$C_6H_4$-O-$(CH_2)_4$-βCD]$_6$. p13

**Figure S21.** 2D NMR HMQC spectrum of $P_3N_3$-[O-$C_6H_4$-O-$(CH_2)_4$-βCD]$_6$. p14

**Figure S22.** 2D NMR COSY spectrum of $P_3N_3$-[O-$C_6H_4$-O-$(CH_2)_4$-βCD]$_6$. p15

**Figure S23.** MALDI spectrum of $P_3N_3$-[O-$C_6H_4$-O-$(CH_2)_4$-βCD]$_6$. p16

**Figure S24.** $^{31}P$-NMR spectrum of $P_3N_3$-[O-$C_6H_4$-O-$(CH_2)_4$-βCD]$_6$. p16

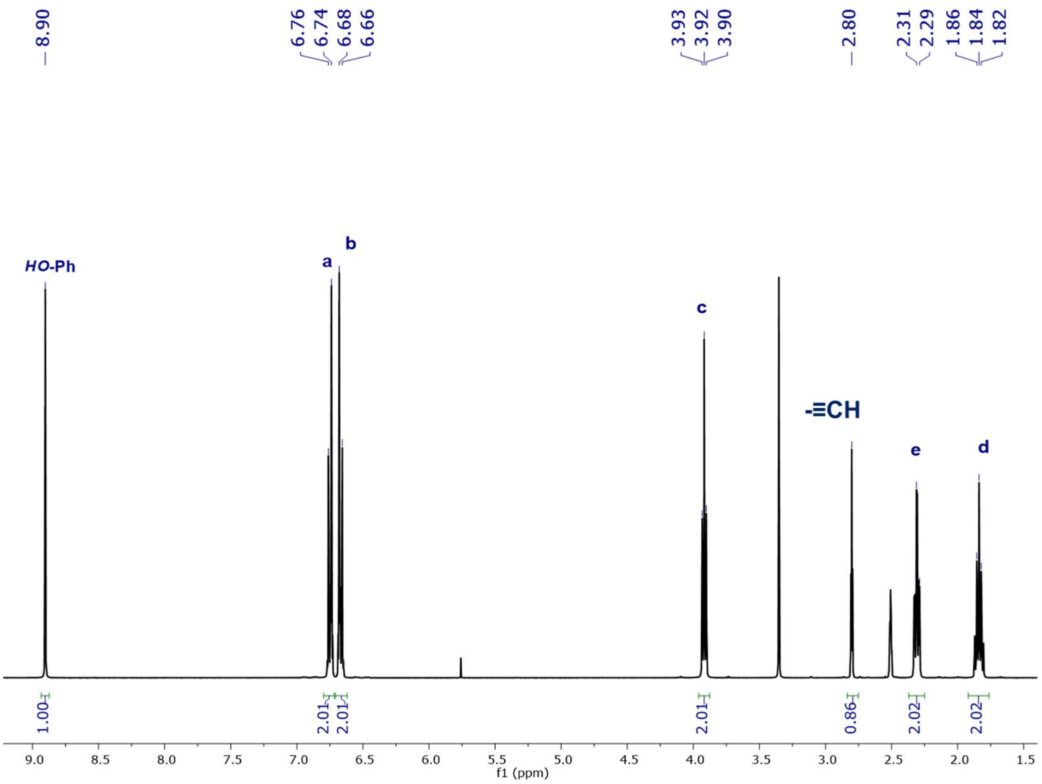


**Figure S1.** $^{1}$H-NMR spectrum of HO-$C_6H_4$-O-$(CH_2)_3$-alkyne.

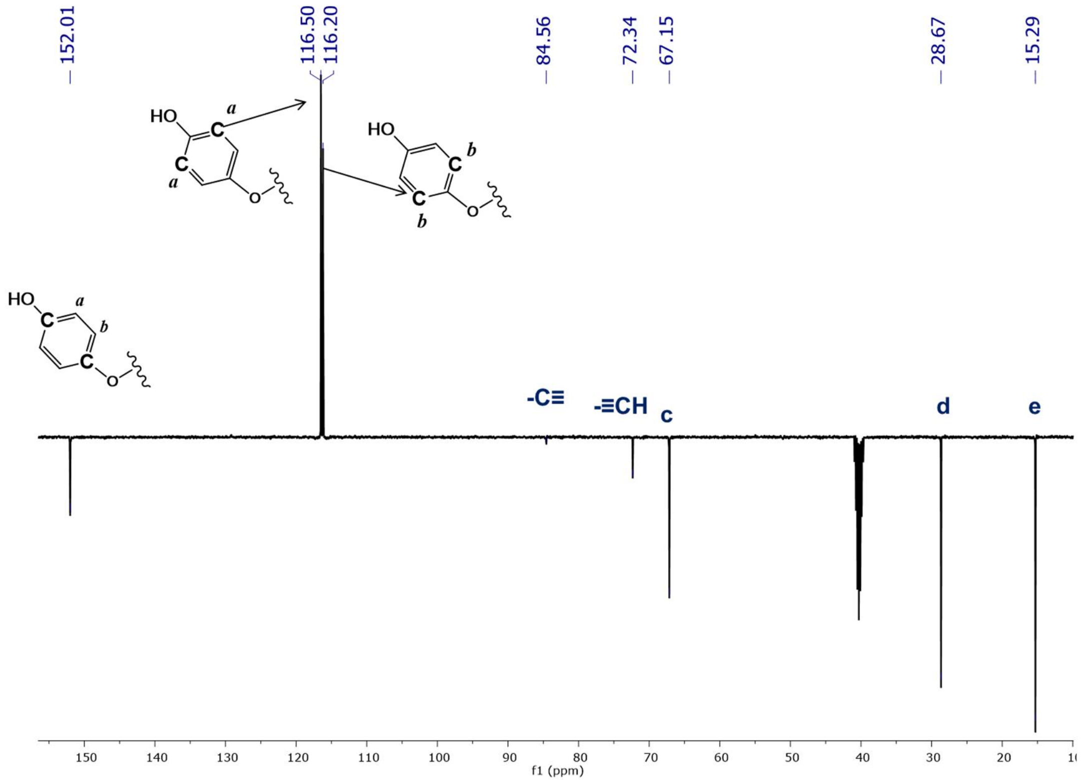


**Figure S2.** $^{13}$C-NMR spectrum of HO-$C_6H_4$-O-$(CH_2)_3$-alkyne.

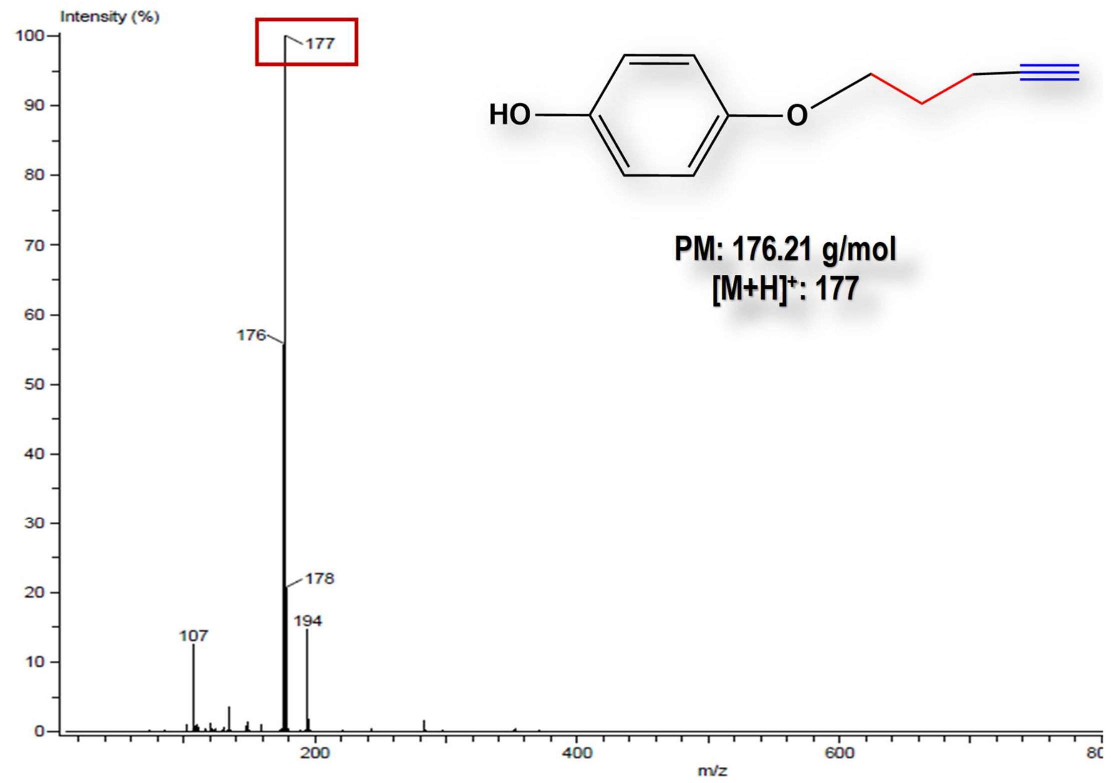


**Figure S3.** DART spectrum of HO-$C_6H_4$-O-$(CH_2)_3$-alkyne.

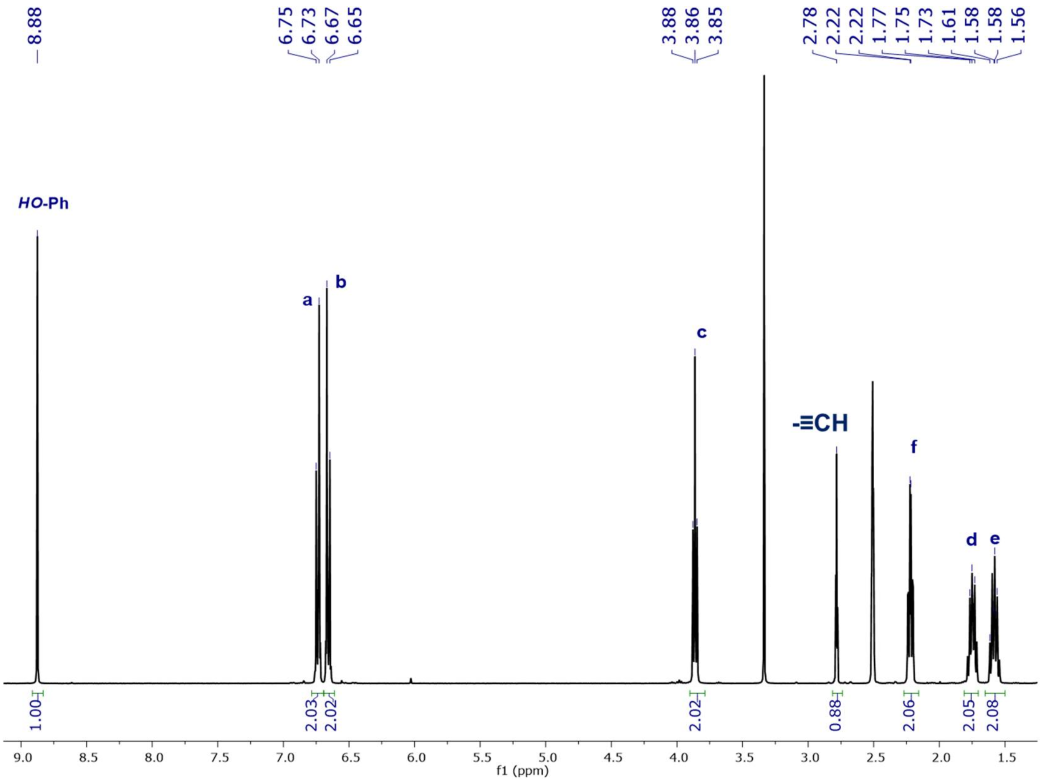


**Figure S4.** $^{1}$H-NMR spectrum of HO-$C_6H_4$-O-$(CH_2)_4$-alkyne.

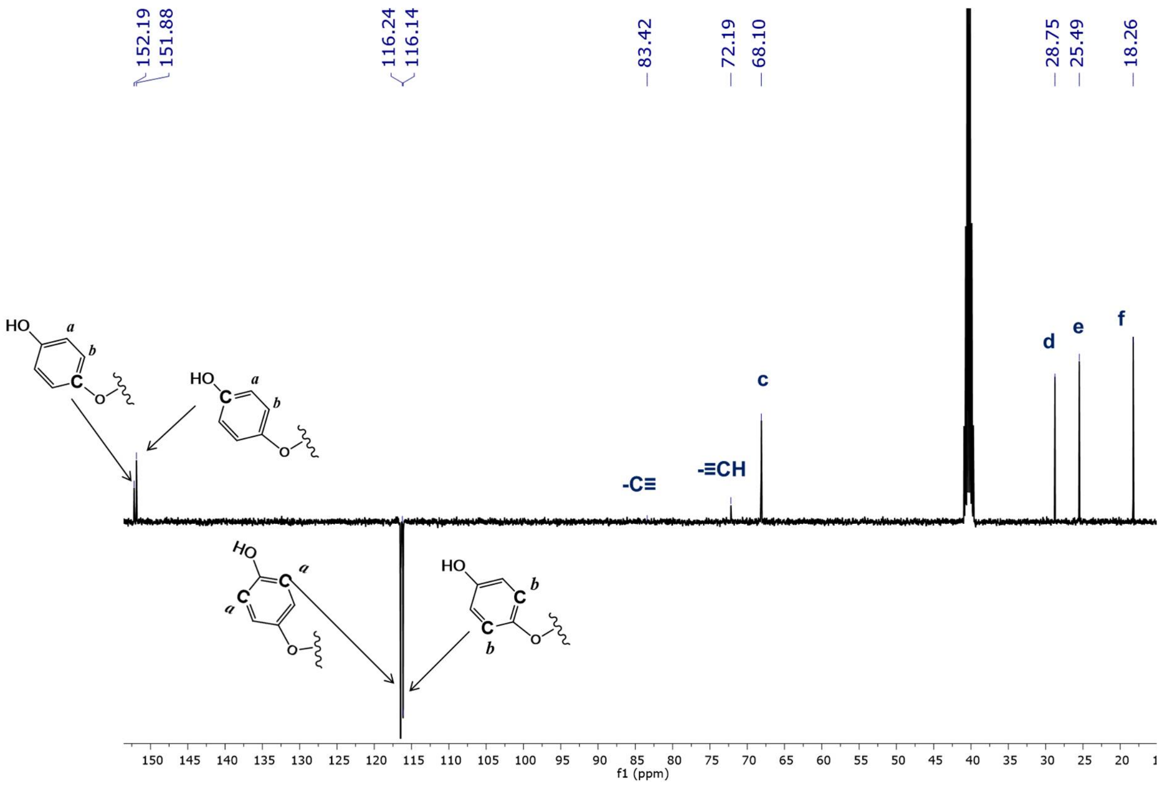


**Figure S5.** $^{13}C$-NMR spectrum of HO-$C_6H_4$-O-$(CH_2)_4$-alkyne.

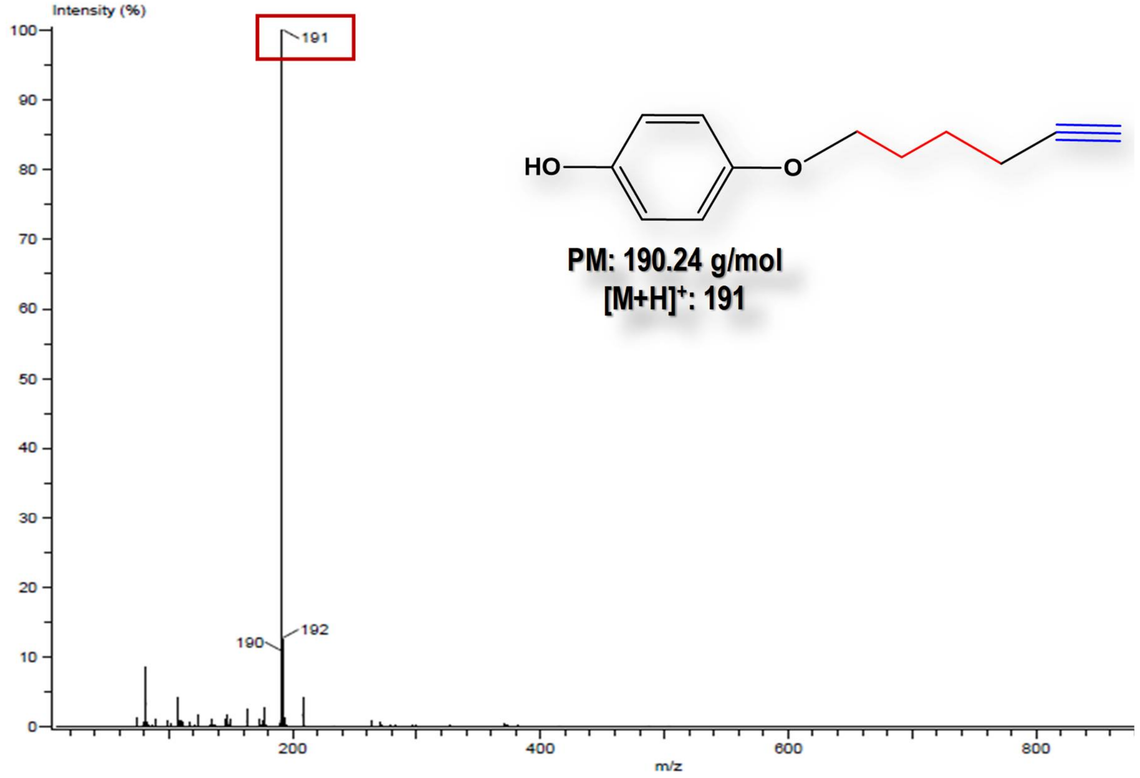


**Figure S6.** DART spectrum of HO-$C_6H_4$-O-$(CH_2)_4$-alkyne.

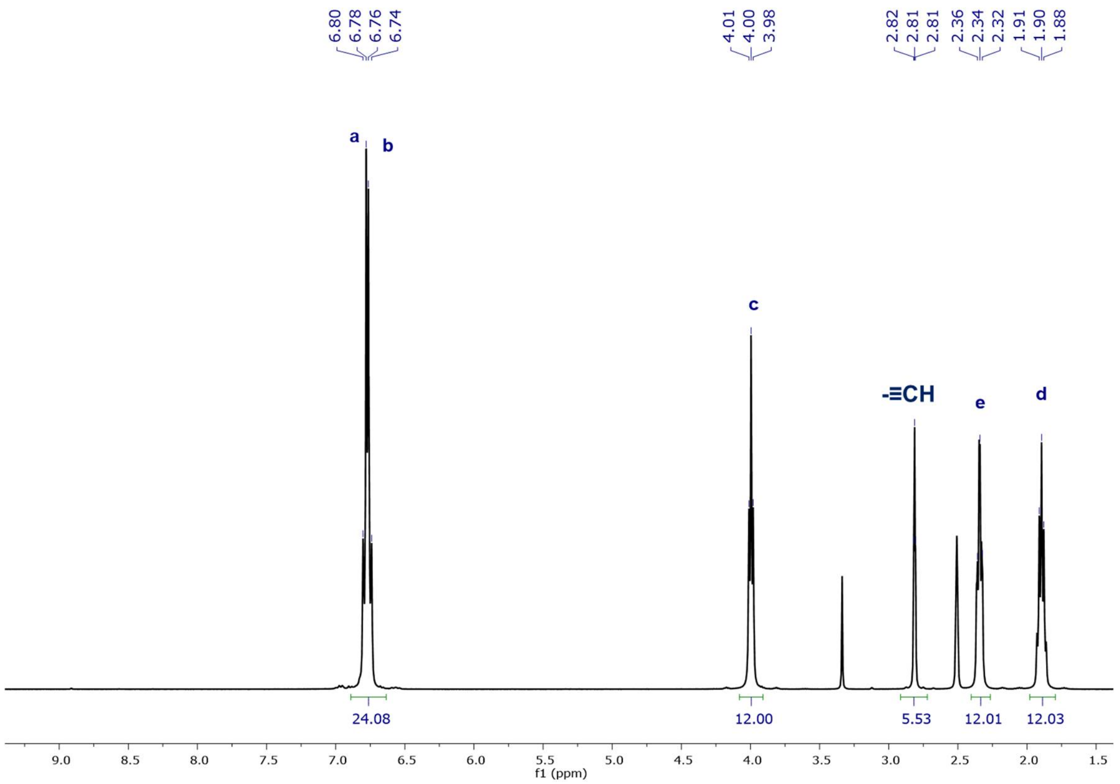


**Figure S7.** $^{1}H$-NMR spectrum of $P_3N_3$-[O-$C_6H_4$-O-$(CH_2)_3$-alkyne]$_6$.

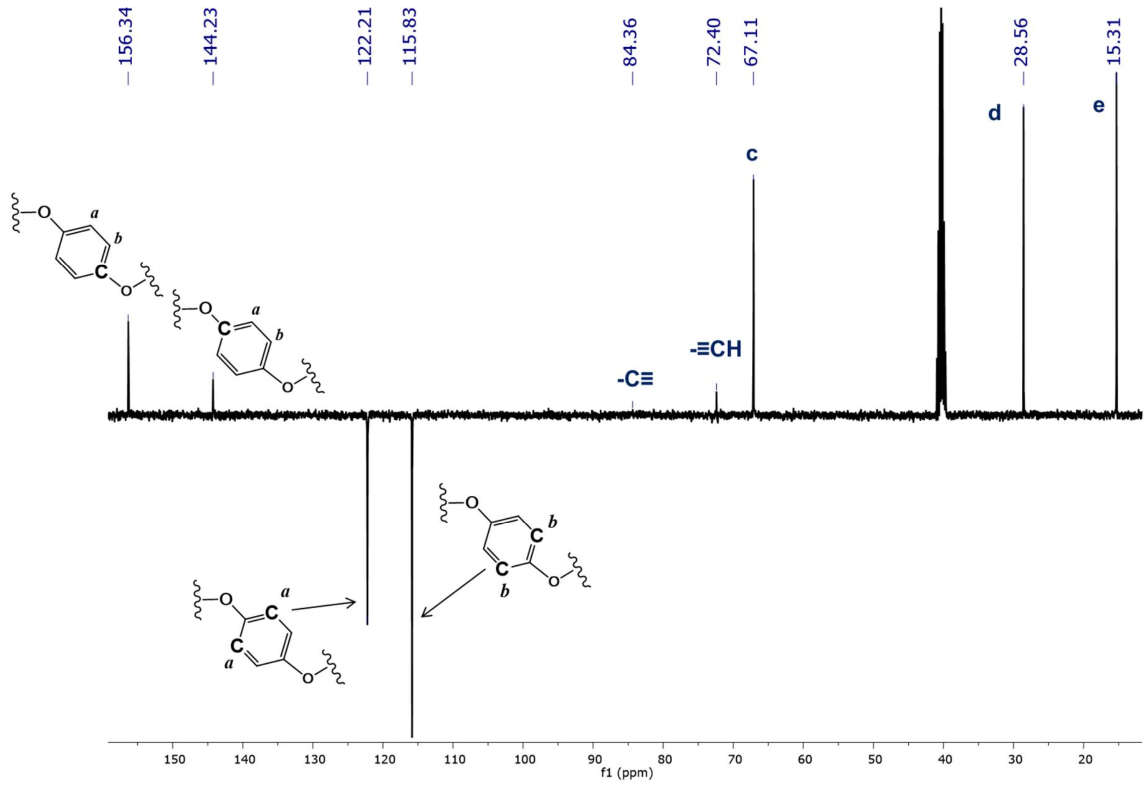


**Figure S8.** $^{13}C$-NMR spectrum of $P_3N_3$-[O-$C_6H_4$-O-$(CH_2)_3$-alkyne]$_6$.

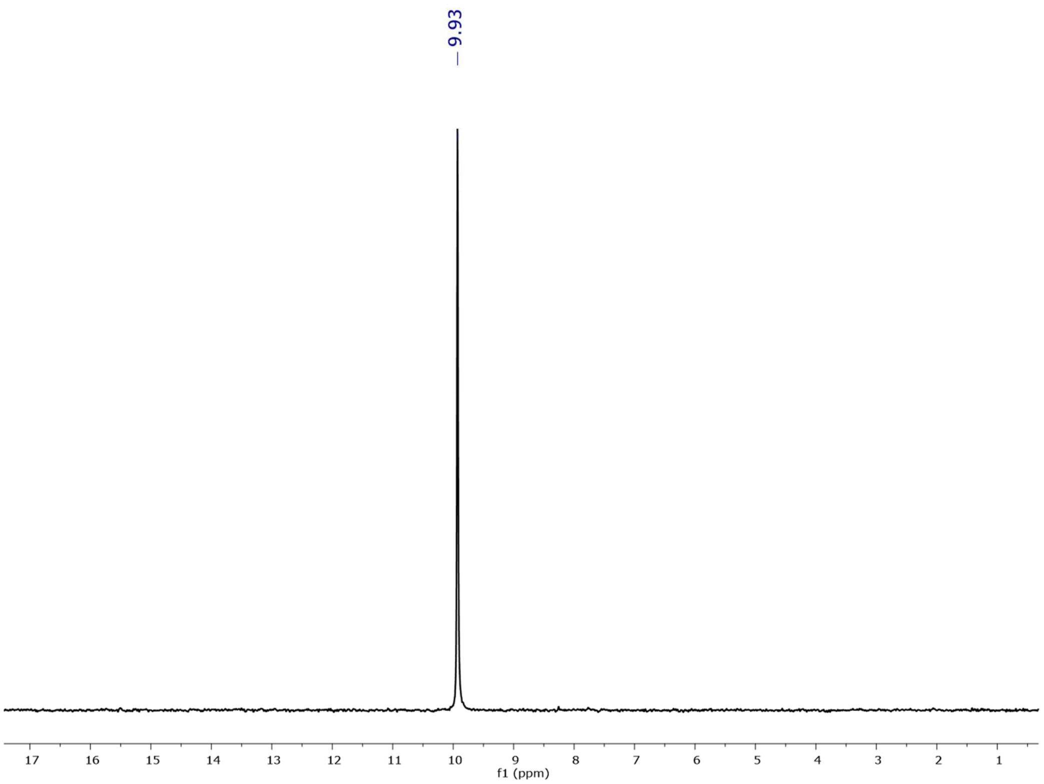


**Figure S9.** $^{31}P$-NMR spectrum of $P_3N_3$-[O-$C_6H_4$-O-$(CH_2)_3$-alkyne]$_6$.

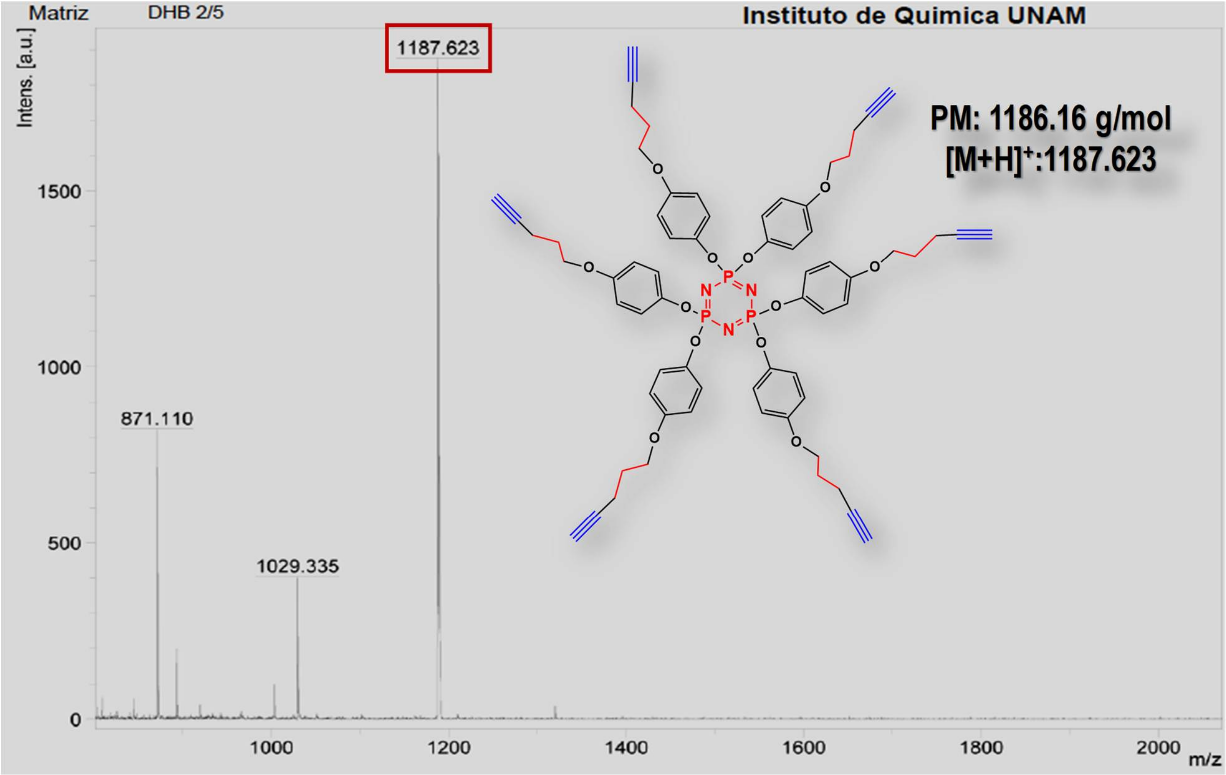


**Figure S10.** MALDI spectrum of $P_3N_3$-[O-$C_6H_4$-O-$(CH_2)_3$-alkyne]$_6$.

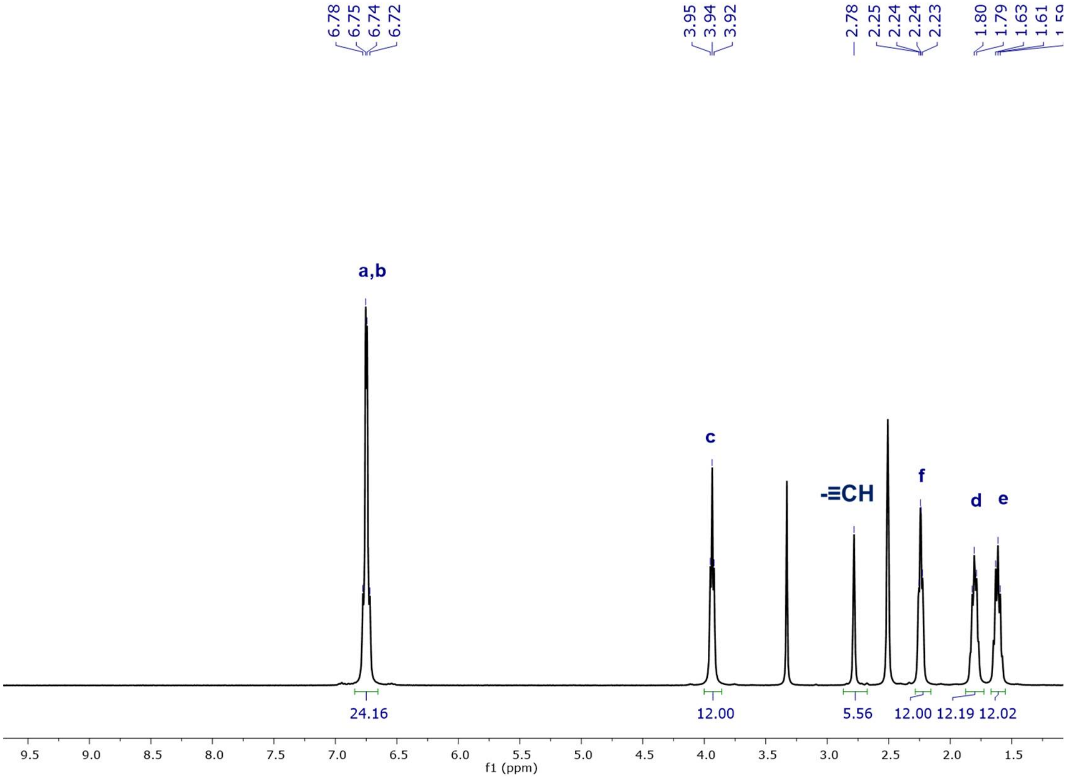


**Figure S11.** $^{1}H$-NMR spectrum of $P_3N_3$-[O-$C_6H_4$-O-$(CH_2)_4$-alkyne]$_6$.

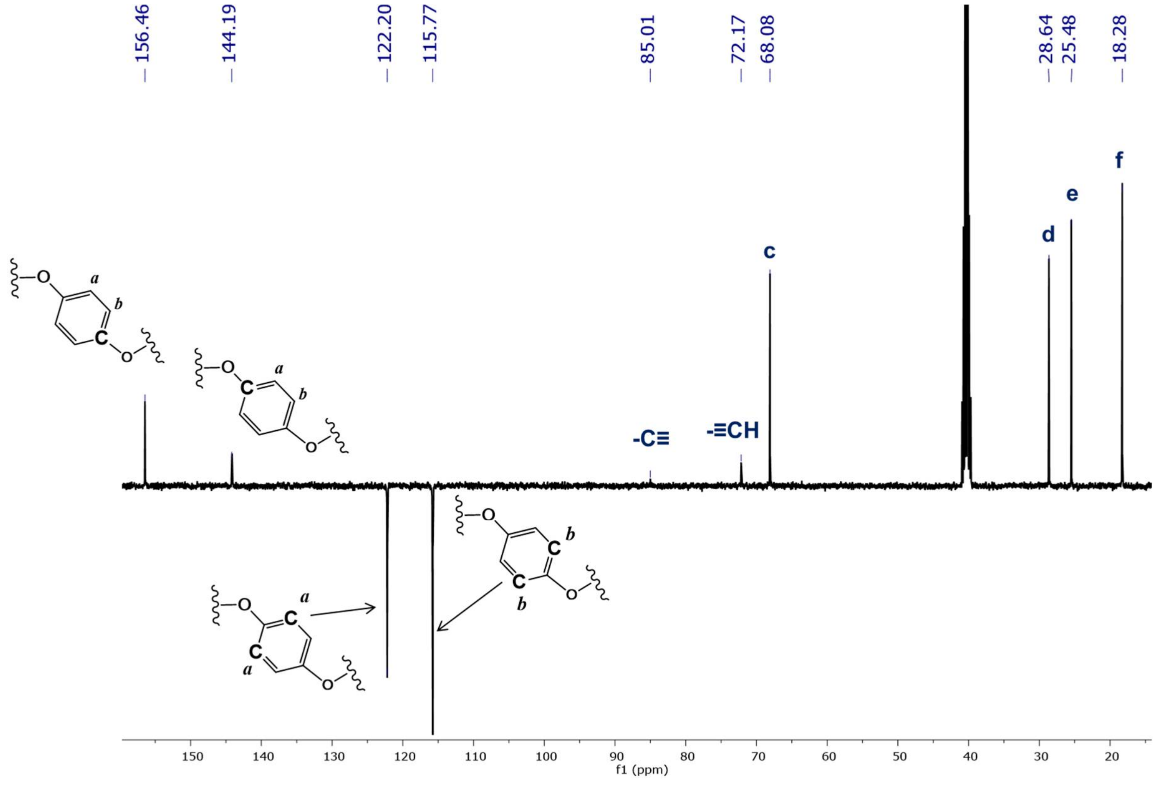


**Figure S12.** $^{13}C$-NMR spectrum of $P_3N_3$-[O-$C_6H_4$-O-$(CH_2)_4$-alkyne]$_6$.

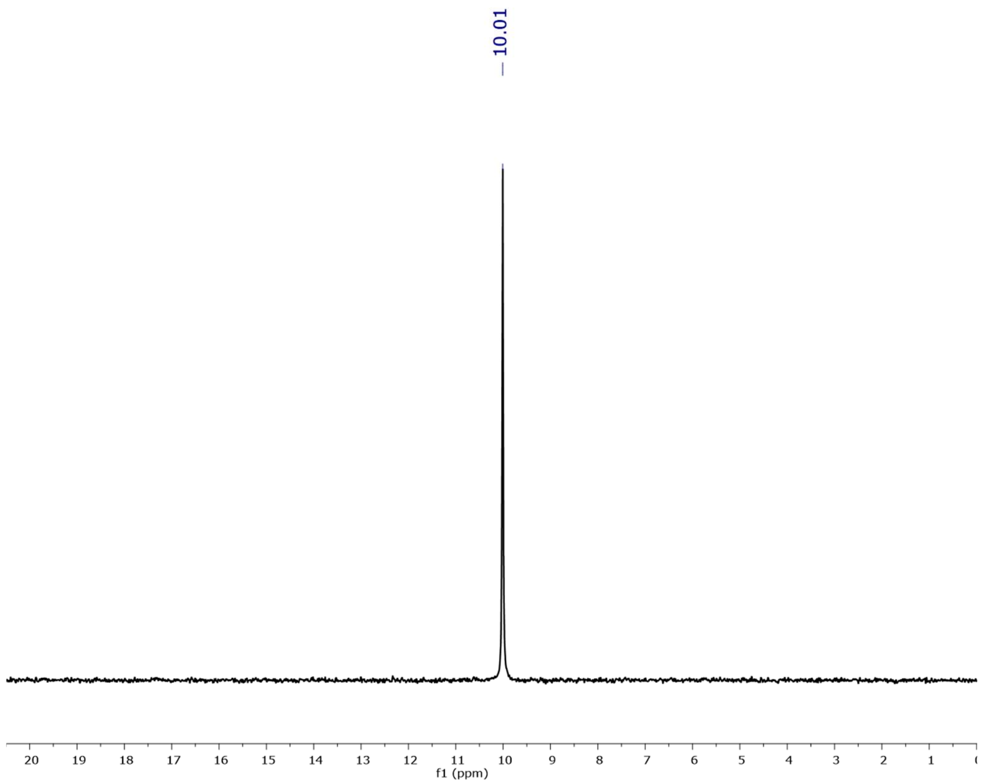


**Figure S13.** $^{31}$P-NMR spectrum of $P_3N_3$-[O-$C_6H_4$-O-$(CH_2)_4$-alkyne]$_6$.

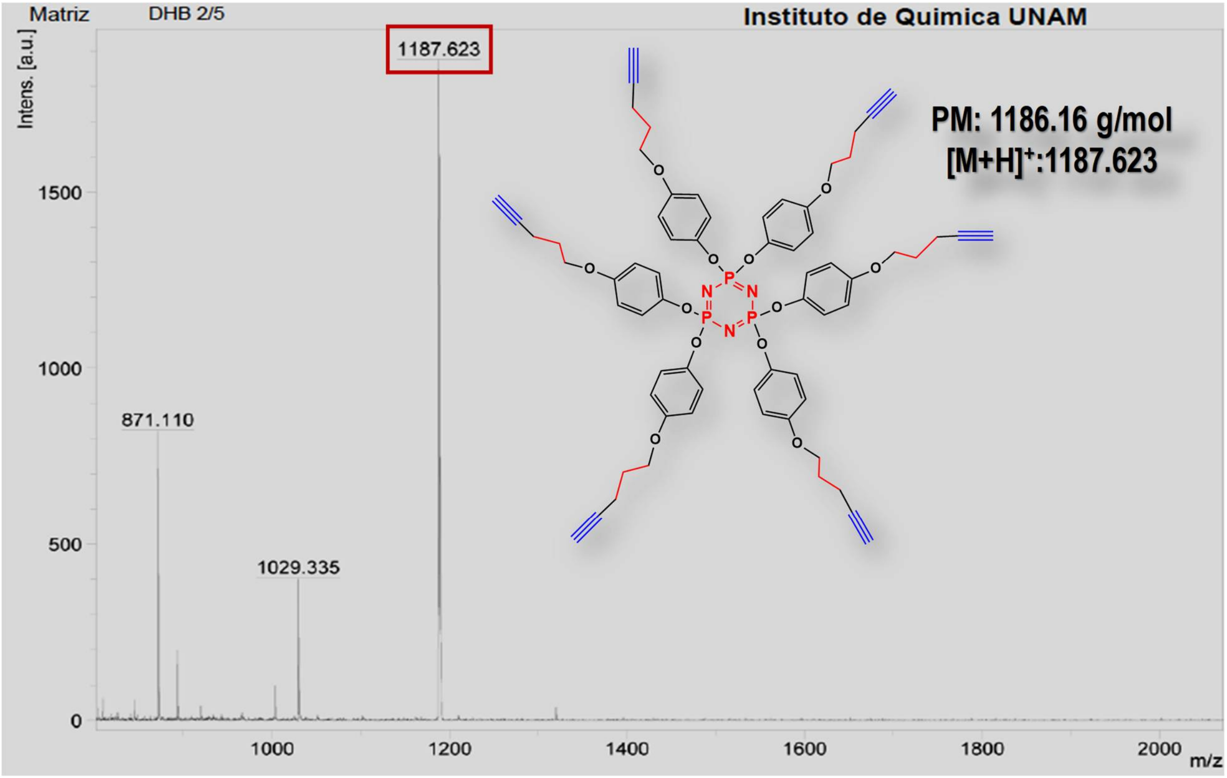


**Figure S14.** MALDI spectrum of $P_3N_3$-[O-$C_6H_4$-O-$(CH_2)_4$-alkyne]$_6$.

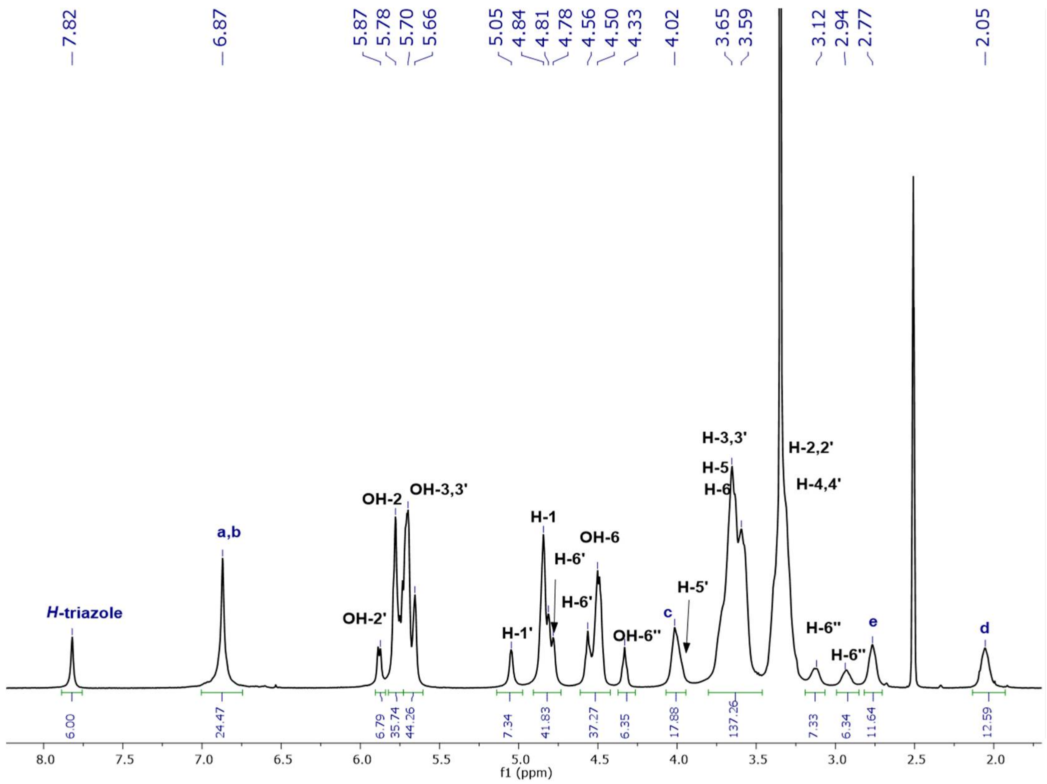


**Figure S15.** $^{1}$H-NMR spectrum of $P_3N_3$-[O-$C_6H_4$-O-$(CH_2)_3$-βCD]$_6$.

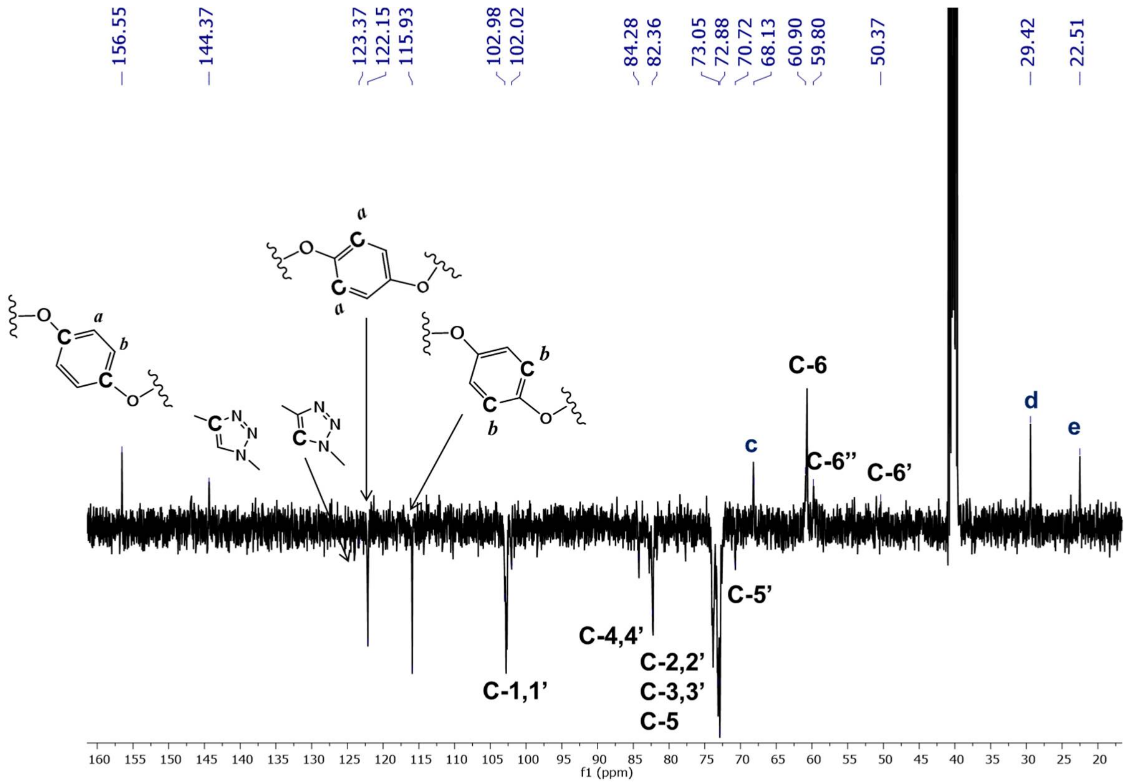


**Figure S16.** $^{13}$C-NMR spectrum of $P_3N_3$-[O-$C_6H_4$-O-$(CH_2)_3$-βCD]$_6$.

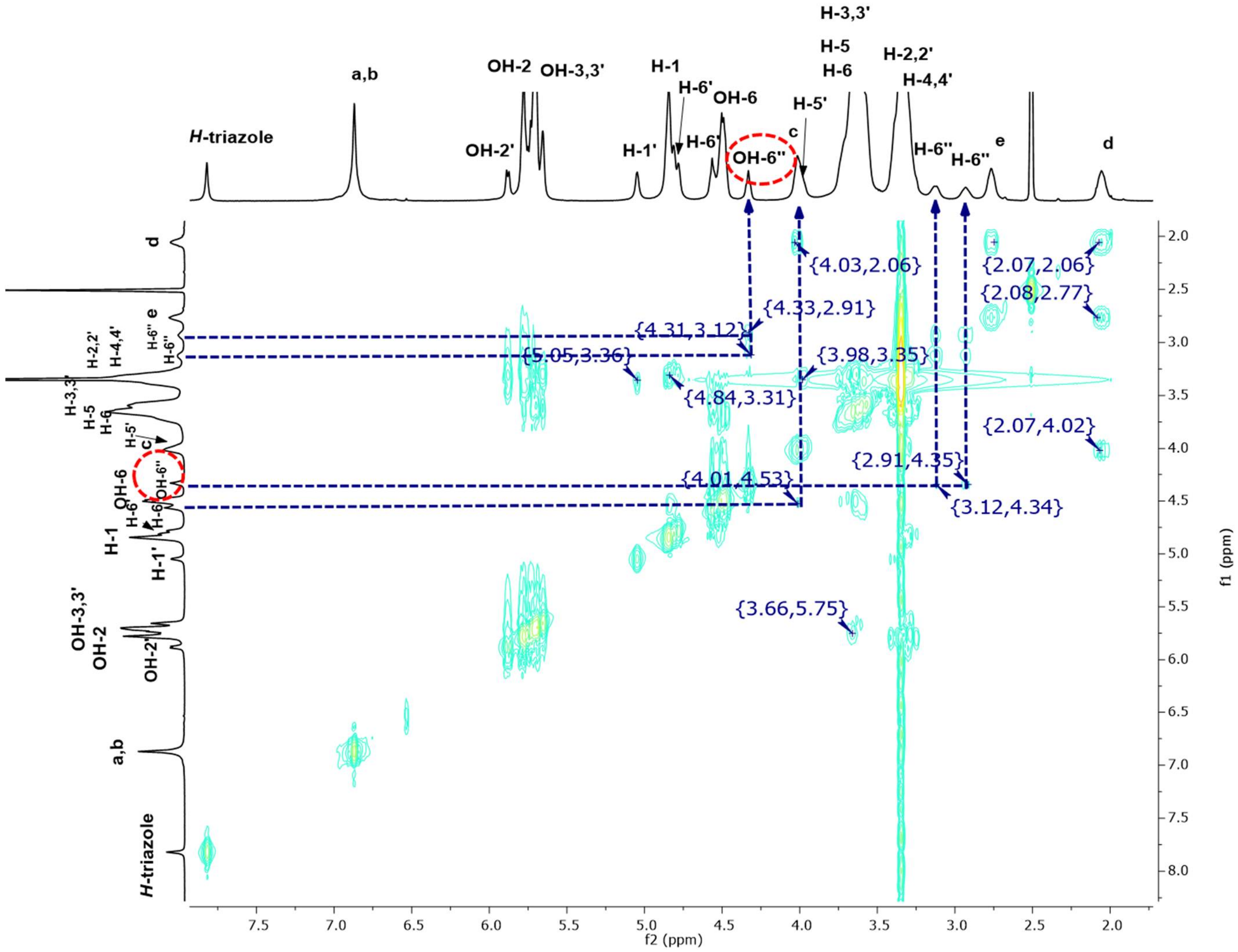


**Figure S17.** 2D NMR COSY spectrum of $P_3N_3$-[O-$C_6H_4$-O-$(CH_2)_3$-βCD]$_6$.

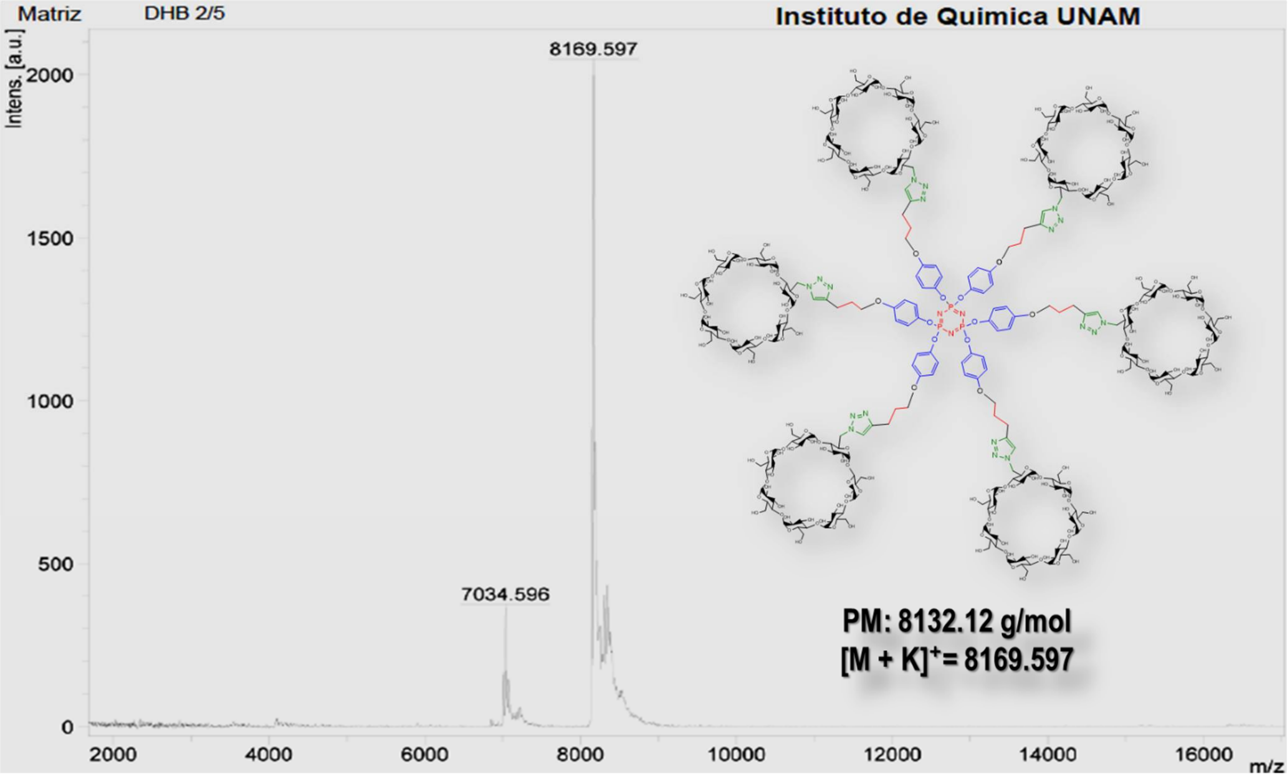


**Figure S18.** MALDI spectrum of $P_3N_3$-[O-$C_6H_4$-O-$(CH_2)_3$-βCD]$_6$.

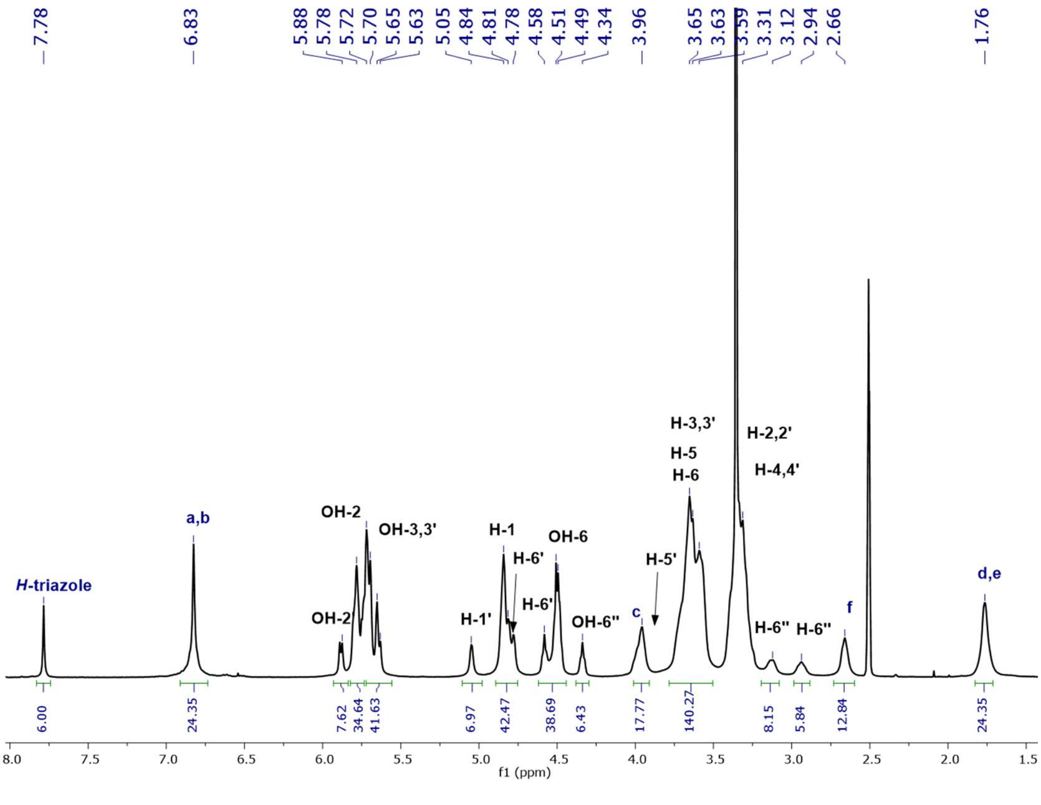


**Figure S19.** $^1$H-NMR spectrum of $P_3N_3$-[O-$C_6H_4$-O-$(CH_2)_4$-βCD]$_6$.

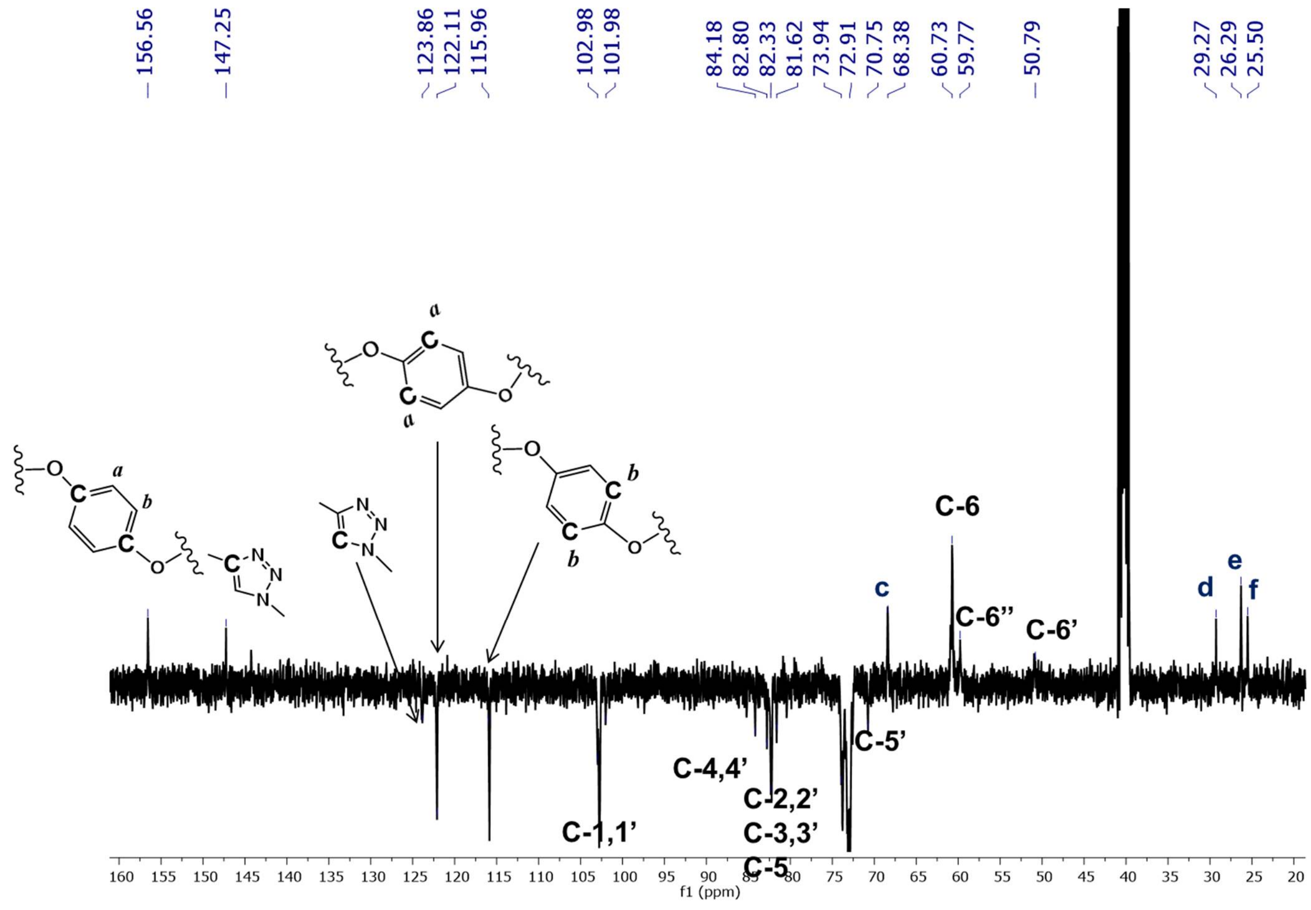


**Figure S20.** $^{13}$C-NMR spectrum of $P_3N_3$-[O-$C_6H_4$-O-$(CH_2)_4$-βCD]$_6$.

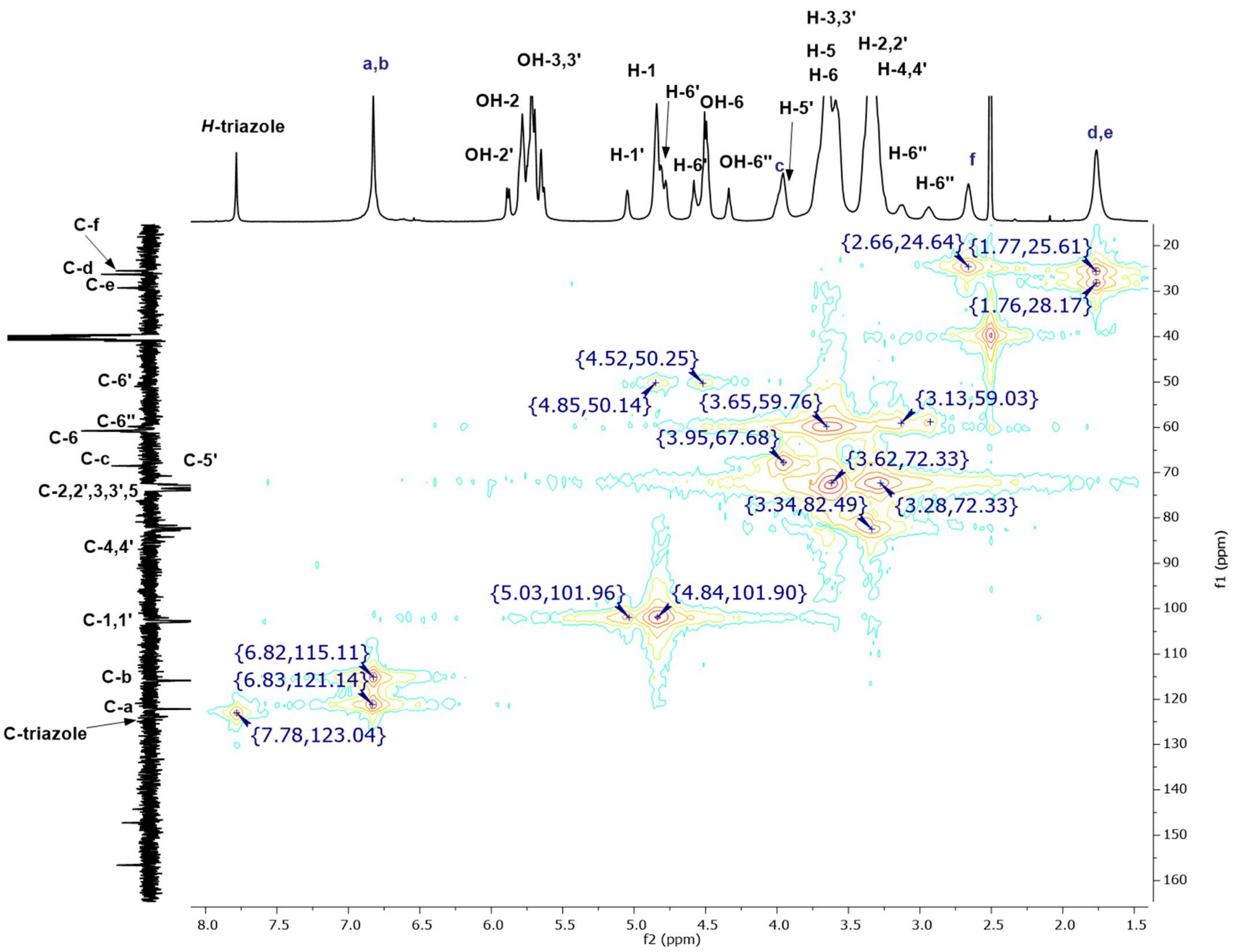


**Figure S21.** 2D NMR HMQC spectrum of $P_3N_3$-[O-$C_6H_4$-O-$(CH_2)_4$-βCD]$_6$.

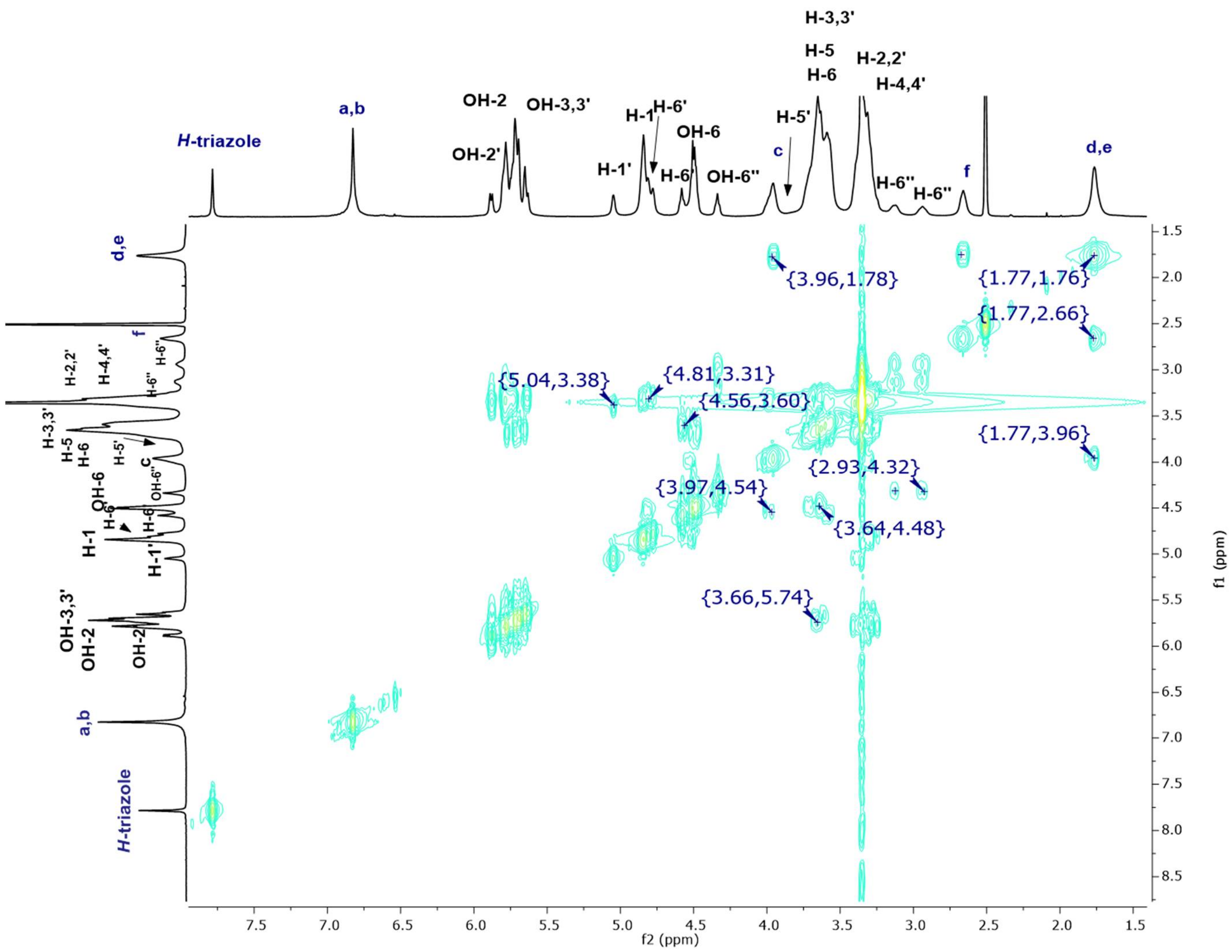


**Figure S22.** 2D NMR COSY spectrum of $P_3N_3$-[O-$C_6H_4$-O-$(CH_2)_4$-βCD$]_6$.

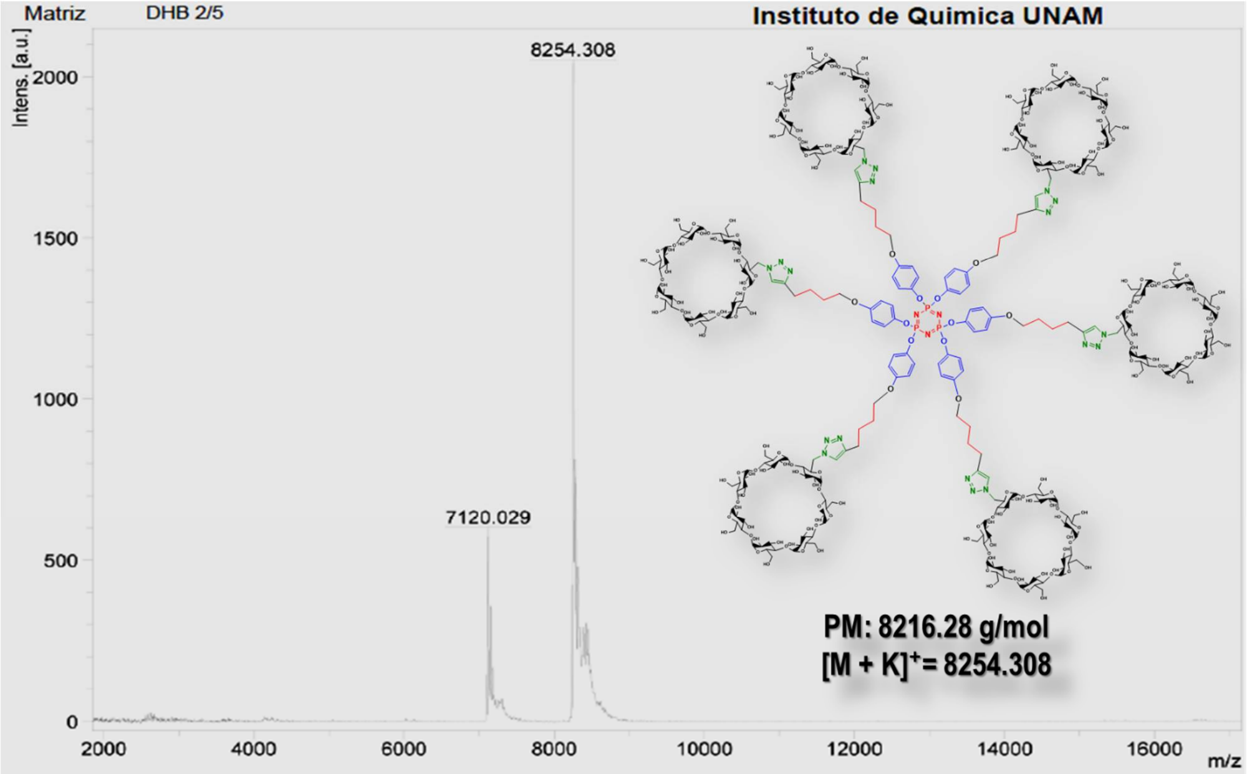


**Figure S23.** MALDI spectrum of $P_3N_3$-[O-$C_6H_4$-O-$(CH_2)_4$-βCD]$_6$.

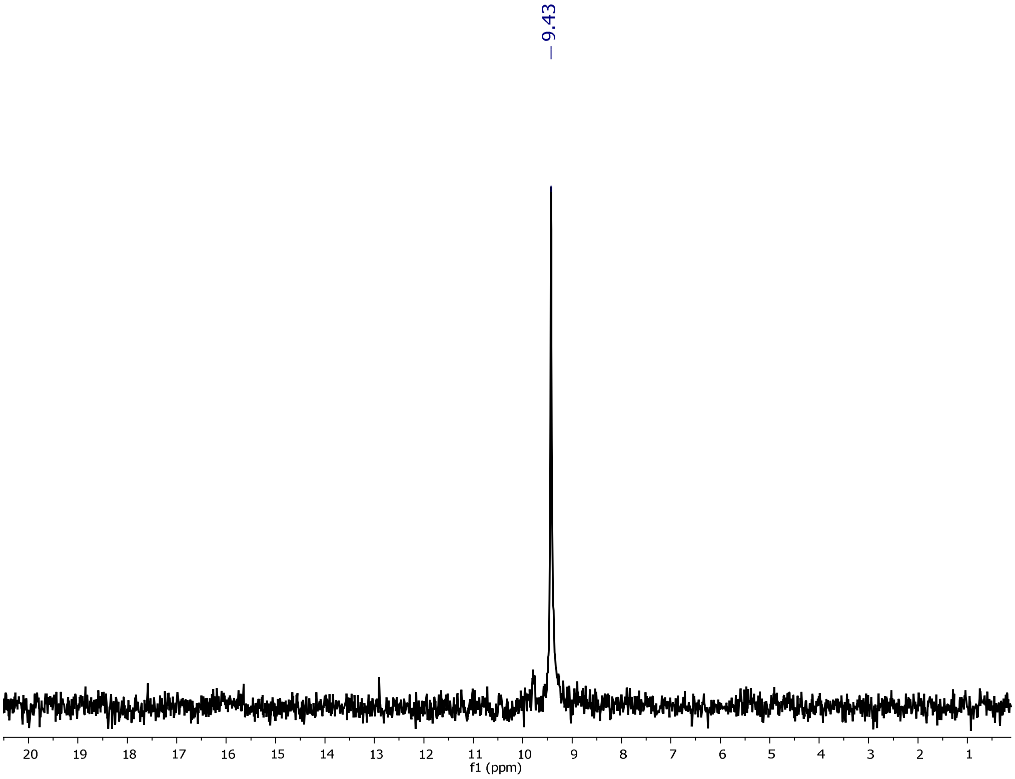


**Figure S24.** $^{31}$P-NMR spectrum of $P_3N_3$-[O-$C_6H_4$-O-$(CH_2)_4$-βCD]$_6$.
